# HMGN2 accelerates the proliferation and cell cycle progression of glioblastoma by regulating CDC20 expression

**DOI:** 10.1016/j.gendis.2024.101433

**Published:** 2024-09-12

**Authors:** Jiacheng Zhong, Shuang Shi, Wen Peng, Hongjuan Cui, Xiaochuan Sun

**Affiliations:** a Department of Neurosurgery, The First Affiliated Hospital of Chongqing Medical University, Chongqing 400016, China; b State Key Laboratory of Resource Insects, Medical Research Institute, Southwest University, Chongqing 400716, China; c Jinfeng Laboratory, Chongqing 401329, China

**Keywords:** CDC20, Epigenetic regulation, Glioma, Histone acetylation, HMGN2

## Abstract

Gliomas represent the most common primary malignant intracranial tumors in adults. Despite recent advances in treatment, the prognosis of patients with glioblastoma remains poor. Epigenetic abnormalities, the hallmarks of various types of cancer, contribute to the dysregulated expression of cancer-related genes. Post-translational modification of histones plays a pivotal role in cancer development and progression by modulating gene transcription, chromatin remodeling, and nuclear structure. Therefore, further exploration of the molecular mechanisms of epigenetic regulation in gliomas and the identification of superior therapeutic targets are required. High-mobility group nucleosomal-binding domain 2 (HMGN2) participates in the epigenetic regulation of genes through histone modification and exhibits significant differential expression between glioma and normal tissues. However, the effect of HMGN2 on gliomas and its underlying mechanisms remain unclear. This study aimed to elucidate these uncertainties by demonstrating that HMGN2 significantly promotes the proliferation of glioma cells. HMGN2 binds to histones and promotes the stability of H3K27ac acetylation in the cell division cycle 20 (CDC20) promoter region, enhancing the transcriptional activity of CDC20 and increasing the proliferation of glioma cells. Moreover, we found that CDC20 expression was negatively correlated with the survival time of patients with glioma. These results suggest that targeting epigenetic regulation, such as the HMGN2/CDC20 axis, may provide a novel direction for the treatment of gliomas.

## Introduction

Glioma is a malignant intracranial tumor derived from glial cells and is the most common primary malignant intracranial neoplasm. The WHO has categorized gliomas into low-grade (WHO grade 1/2) and high-grade gliomas (WHO grade 3/4) based on the degree of malignancy. Glioblastoma multiforme (GBM) has the highest degree of malignancy with the poorest prognosis and survival rates. Presently, with continuous advances in diagnostics and treatment modalities, patients with newly diagnosed GBM undergo a standard treatment protocol, including the maximum extent of surgical resection followed by the Stupp regimen (concurrent radio-chemotherapy with temozolomide and six cycles of adjuvant temozolomide chemotherapy).^1^, ^2^, ^3^ Despite these efforts, the median overall survival for these patients remains a mere 14.6 months.^3^^,^^4^ Even with the addition of tumor-treating fields (TTFields), an emerging physiotherapy combined with temozolomide, the median overall survival is only 20.9 months.^5^, ^6^, ^7^ Hence, identifying more effective therapeutic diagnostic targets to improve patient outcomes remains an elusive goal in GBM research.

In recent decades, accumulating evidence has illuminated the fundamental role of epigenetic alterations in tumorigenesis and malignant progression.^8^ Histone modification is one of the most used epigenetic methods for regulating chromatin structure, DNA repair, and gene expression. Previous studies have concluded that dysregulations of histone modification were observed in glioma. The histone modification reprogramming manipulates gene expression, such as HOXA1 (homeobox A1) and P21, regulates GBM cell proliferation, migration, and invasion, and inhibits cell apoptosis.^9^^,^^10^ Besides, the overexpression of histone modification enzymes such as PCR2 (polycomb repressive complexes 2) mediates immunosuppression by blocking the expression of immune-stimulatory cytokines in glioma.^11^ HMGN2 (high-mobility group nucleosomal-binding domain 2) is a member of the high-mobility group (HMG) that binds to nucleosomes and is enriched in epigenetic marks of active chromatin, especially in H3K27ac-modified histones.^12^ Several studies have confirmed that the binding of HMGNs to nucleosomes reduces chromatin compaction and promotes the growth and metastasis of different tumors by increasing accessibility and locally enhancing the transcriptional activity of oncogenes, shortening the lifespan of experimental animals. Subsequently, some studies have reported that HMGN2 is essential for prolactin-induced CISH (cytokine-inducible SH2 containing protein) expression by promoting histone H1 loss and STAT5 (signal transducer and activator of transcription 5) binding in breast cancer.^13^, ^14^, ^15^ In gastric cancer, HMGN2 is significantly associated with recurrence-free survival. The expression of HMGN2 correlates with Th2 cells and T helper cells, suggesting that it may play a role in tumor immune escape.^16^ However, the opposite effect has been observed in osteosarcoma^17^ and cervical cancer.^18^ In the current study, to elucidate the role of HMGN2 in glioma, we conducted a bioinformatics analysis which revealed significant up-regulation of HMGN2 expression in gliomas versus normal brain tissue. Furthermore, HMGN2 expression positively correlated with glioma malignancy grade and poorer patient prognosis.

Under these circumstances, we conducted a bioinformatics analysis using public data to elucidate the molecular mechanisms of HMGN2 in glioma cells. Preliminary results indicate that HMGN2 promotes tumor cell progression by regulating the cell cycle. Errors in chromosome segregation are widespread in human cancers.^19^ The spindle assembly checkpoint is crucial during the cell cycle as it ensures the correct segregation of chromosomes in anaphase. To prevent potential errors during anaphase onset, spindle assembly checkpoint inhibits the ubiquitylation of cyclin B1 and securin by blocking the activation of the anaphase-promoting complex/cyclosome (APC/C) ubiquitin ligase.^20^, ^21^, ^22^ CDC20 (cell division cycle 20) is a critical activator of APC/C and its stable expression is particularly important for maintaining normal cell mitosis.^23^ Previous studies have shown that CDC20 is up-regulated in a variety of malignant tumors such as breast cancer, pancreatic cancer, lung cancer, and hepatocellular carcinoma, resulting in a poor prognosis.^24^, ^25^, ^26^, ^27^, ^28^ Furthermore, recent findings indicated that CDC20 may represent gene signatures as therapeutic, diagnostic, and prognostic prediction targets. These findings prompted us to investigate the role of CDC20 in glioma.

In this study, we confirmed that HMGN2 regulated GBM proliferation both *in vitro* and *in vivo* by influencing the mitotic phase of the cell cycle. Furthermore, we discovered that HMGN2 increased and stabilized the acetylation level of H3K27 by binding to histone 3, thereby enhancing chromatin activity and promoting CDC20 protein expression. This study reveals the epigenetic regulatory function of HMGN2, showing how it manipulates the transcriptional activity of proliferation-related genes to accelerate cell cycle progression in GBM cells. Taken together, these results suggest that HMGN2 could serve as a prognostic indicator and a new therapeutic target for gliomas.

## Materials and methods

### Data acquisition and correlation analysis

RNA sequencing data and clinical data of glioma patients were downloaded and collated from The Cancer Genome Atlas (TCGA) database. According to the TCGA database, we divided the expression of HMGN2 in glioma patients into low-expression and high-expression groups, and R package “DESeq2” was used to filter differentially expressed genes^29^ (DEGs, *P*.adj < 0.05, |log_2_ Fold Change| > 1.5). The DEGs derived from the HMGN2 high and low groups were used for Gene Ontology (GO) and Kyoto Encyclopedia of Genes and Genomes (KEGG) pathway enrichment analyses. The DEGs annotated in the GO database were classified into GO functions using the ClusterProfiler package,^30^ while the DEGs for KEGG enrichment analysis were mapped to the KEGG database.^31^ After the enrichment analysis, the top 10 KEGG pathways and the top 8 terms in each GO category were selected. Gene set enrichment analysis (GSEA) was performed on the DEGs enriched in the cell cycle pathway.^32^

### Cell lines and clinical samples

The LN229, U-87 MG, A172, U118, and U251 cell lines used in all experiments were purchased from the American Type Culture Collection (Manassas, VA, USA). Patient-derived glioma stem cell line TS543 cells were provided by Dr. Fanghui Lu (Chongqing Medical University). LN229, U-87 MG, A172, U118, and U251 cells were cultured as previously described. The glioma stem cells were cultured in human NSC Maintenance Media (Millipore, Shanghai, China), supplemented with EGF (epidermal growth factor) and FGF-β (transforming growth factor-β) (20 ng/mL each). Prior approval for the treatment of clinical tissue samples was obtained from the Ethics Committee of The First Affiliated Hospital of Chongqing Medical University (Chongqing, China). Informed consent was obtained from all the patients involved in the study.

### Plasmid and lentivirus preparation and infection

HMGN2-shRNA#1/#2/#3 and scrambled shRNA were purchased from Sangon (Shanghai, China) and cloned into the pLKO.1 vector, which was obtained from Sigma–Aldrich (Shanghai, China). The HMGN2 OE (overexpression), HMGN2-3XFlag, HMGN2-ΔNLS-3XFlag, and CDC20 OE plasmids were obtained from Youbao Corporation (Changsha, China). Lentiviruses were prepared using Lipofectamine 2000 (Invitrogen, Carlsbad, CA, USA) to cotransfect the shRNA plasmids along with three packaging plasmids (pLP1, pLP2, pLP/VSVG) into HEK293FT cells over 48 h. Subsequently, GBM cells underwent lentiviral infection twice in the presence of polybrene at a final concentration of 4 μg/mL. Following infection, the cells were screened with a concentration of 1 μg/mL puromycin for subsequent experimental procedures.

### mRNA isolation and qPCR analysis

mRNA was isolated from GBM cells using RNAiso Plus (Takara, Dalian, China). mRNA was reverse-transcribed into cDNA using a cDNA synthesis kit according to the manufacturer's instructions (Promega, Beijing, China). Quantitative polymerase chain reaction (qPCR) was performed using SYBR Green Master Mix (Thermo Scientific, Shanghai, China), and GAPDH was used as an internal normalization control gene. The ΔΔCt method was applied for the qPCR analysis. Primer sequences used for qPCR are listed in Table S1.

### Immunohistochemical staining

The paraffin-embedded tissue sections were deparaffinized, hydrated, and subjected to antigen retrieval. Subsequently, the treated tissue sections were incubated with primary antibodies HMGN2 (Abcam, Shanghai, China), CDC20 (Proteintech, Wuhan, China), and Ki67 (AiFang biological, Hunan, China) overnight at 4 °C, followed by incubation with horseradish peroxidase-conjugated secondary antibodies at room temperature for 1 h. Then sections were incubated with diaminobenzidine to visualize the staining. Subsequently, they were counterstained with hematoxylin and were then thoroughly examined under a microscope.

### Immunofluorescence staining

Cell slides were fixed with 4% paraformaldehyde at room temperature for 15 min, and washed with phosphate buffer saline solution (PBS; Vazyme, Nanjing, China). Then the cells were blocked and permeabilized with 5% BSA/0.1% TritonX-100/PBS at room temperature for 1 h. After removal of the blocking buffer, the tissue was incubated with the primary antibody mix HMGN2 (Abcam, Shanghai, China) and Flag (Proteintech, Wuhan, China) at 4 °C overnight. After three times of washing (5 min each) with 0.2% Tween in PBS on a shaker at room temperature, the slides were incubated with the secondary antibody mix and DAPI at room temperature for 1 h. The tissue was then washed three times (5 min each) with 0.2% Tween-20 in PBS on a shaker at room temperature and then covered with a coverslip.

### Western blotting and co-immunoprecipitation

Western blotting and co-immunoprecipitation were performed as described previously.^33^

### Flow cytometry for cell cycle assays

For the cell cycle assay, cells were fixed for 48 h with 75% ethanol at 4 °C and incubated with a propidium iodide solution containing RNase at 37 °C for 30 min. According to the manufacturer's protocols, flow cytometry (CytoFLEX, BECKMAN COULTER) was used to analyze cell cycle distribution.

### Cell viability and proliferation assays

For the cell counting kit 8 (CCK8) assay, 1 × 10^3^ GBM cells per well were cultured in 96-well plates for three days. The CCK8 assay was performed by adding 20 μL of CCK8 solution (Life-lab, Shanghai, China) and incubating the cells for 2 h. The absorbance of the wells was measured at 450 nm. For the EdU incorporation assay, 3 × 10^4^ cells were seeded into 24-well plates. The cells were incubated with EdU (Sigma) for 2 h, fixed, permeabilized, blocked, incubated with antibodies, and stained. The positive EdU incorporation rate was calculated from at least five random microscopic fields of view.

### Chromatin immunoprecipitation (ChIP)

The ChIP assay was performed using a ChIP kit (Millipore). LN229 and HEK293FT cells were crosslinked with 1% fresh formaldehyde and lysed using SDS lysis buffer. DNA was ultrasonically sheared into 200–800 bp fragments, and the precleared chromatin was immunoprecipitated using a ChIP-grade antibody. After reverse crosslinking, the purified DNA was used for qPCR. The primer sequences used for ChIP-qPCR are listed in Table S1.

### Animal experiments

Nude mice (NOD/SCID, female) were obtained from the Beijing Experimental Animal Research Center (Beijing, China). To establish intracranial xenografts, 1 × 10^5^ treated GBM cells were intracranially injected into mice via an *in-situ* injection system. Randomization and single-blinding were used for the measurements. The mice were observed daily for signs of disease progression and euthanized upon reaching a moribund state. The brains of mice were removed and fixed with 4% paraformaldehyde. All the experimental protocols were conducted by the regulations of the Animal Protection and Use Committee of Chongqing Medical University and the Guide for the Protection and Use of Laboratory Animals (Ministry of Science and Technology of China, 2006). After collection and fixation of cell line-derived xenograft tumor samples, they were embedded and stained with hematoxylin and eosin. Following full-section scanning of the largest tumor surface, the tumor area was measured using the ImageJ software.

### Patient data analysis

Prognostic and gene expression data were downloaded from the Gene Expression Profiling Interactive Analysis (GEPIA) database (http://www.cgga.org.cn), Chinese Glioma Genome Atlas (CGGA; http://www.cgga.org.cn), TCGA (https://portal.gdc.cancer.gov). The related database algorithm determined the critical value for the separation of the high- and low-expression groups.

### Statistical analysis

The results were expressed as mean ± standard deviation. All experiments were performed independently at least three times. Statistical significance was evaluated using either student's *t*-test or ANOVA, with a *P*-value <0.05 considered as statistically significant. Analysis of HMGN2 expression in glioma samples in the databases was performed using the Kruskal–Wallis non-parametric test. Survival curves were analyzed using log-rank and Wilcoxon tests. GraphPad Prism 5 (GraphPad Software, Inc., San Diego, CA, USA) was used for data analysis.

## Results

### Elevated HMGN2 expression in gliomas is closely associated with increased tumor grade and poor prognosis

**Figure 1:**
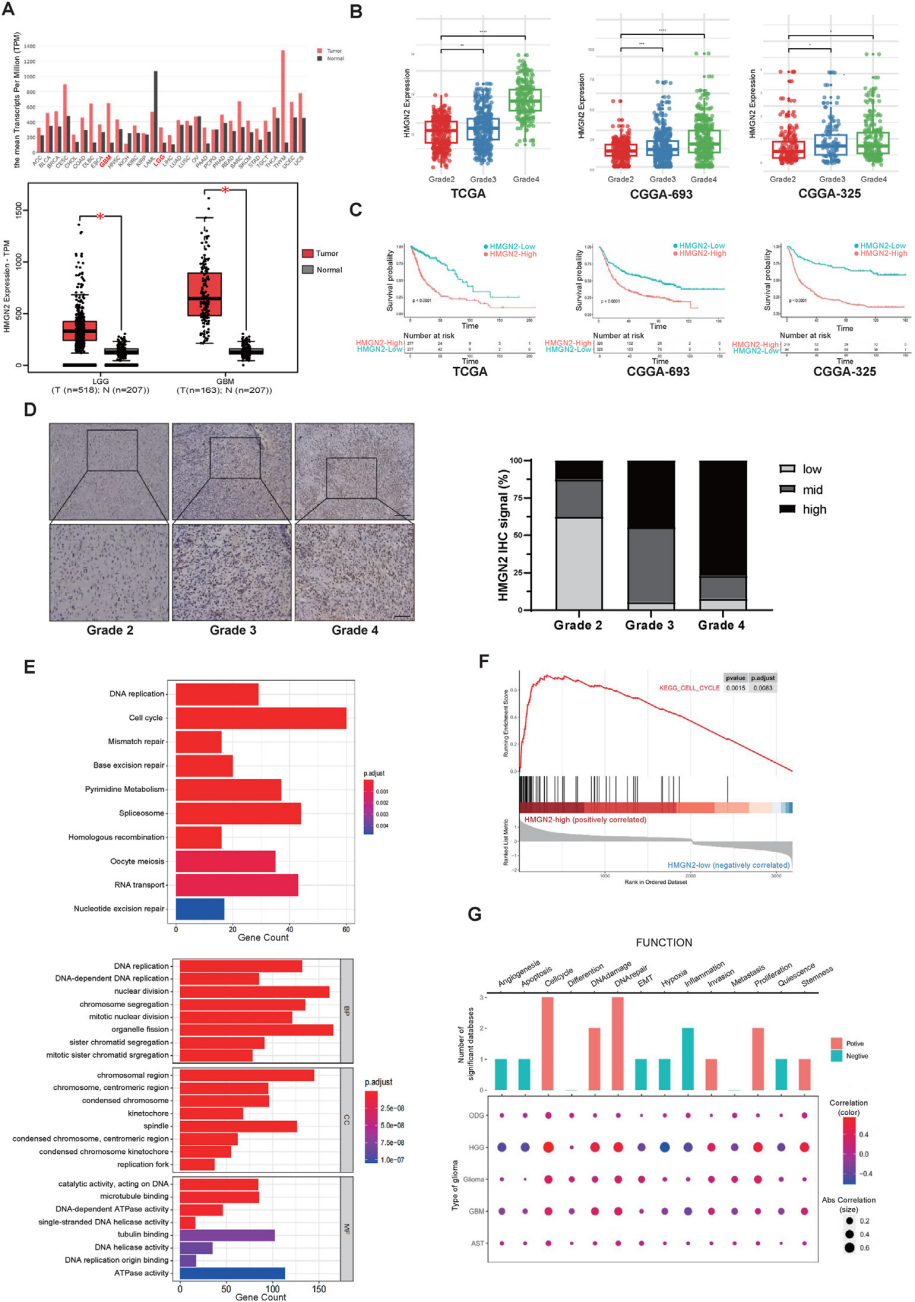
High expression of HMGN2 associated with poor prognosis in patients with glioma. **(A)** Differences in the expression level of the HMGN2 (mRNA sequencing) in different cancers versus adjacent normal tissues analyzed by GEPIA. The glioma samples, low-grade gliomas (LGG), and glioblastoma multiforme (GBM) are labeled in red/bold. **(B)** Analysis of HMGN2 expression in different grades of glioma via the TCGA, CGGA-693, and CGGA-325 databases. **(C)** Analysis of the prognostic significance of HMGN2 in glioma via the TCGA, CGGA-693, and CGGA-325 databases. **(D)** Immunohistochemical staining was performed to evaluate HMGN2 expression in glioma tissue samples (grade 2, *n* = 16; grade 3, *n* = 18; grade 4, *n* = 26). Scale bar for the image above, 100 μm; scale bar for the image below, 50 μm. **(E)** The KEGG and GO enrichment analyses were based on the differentially expressed genes associated with HMGN2. **(F)** GSEA analysis of differentially expressed genes enriched in KEGG_CELL_CYCLE genes set. **(G)** Relevance of HMGN2 across 14 functional states in different types of gliomas.

To explore HMGN2's potential pro-tumor or anti-tumor effect, we first used GEPIA (http://gepia.cancer-pku.cn/) to analyze its expression pattern in gliomas. It showed that HMGN2 expression was up-regulated in gliomas compared with normal brain tissue, with GBM (*n* = 163) exhibiting a more pronounced increase than low-grade gliomas (*n* = 518) (Fig. 1A). We subsequently analyzed mRNA levels according to data from the TCGA and CGGA (mRNAseq 693 and mRNAseq 325) databases. We found that the mRNA level of HMGN2 was significantly increased in high-grade gliomas compared with that in low-grade gliomas in the TCGA and CGGA datasets (Fig. 1B). This led to the conclusion that HMGN2 expression was positively associated with the grade of malignancy in gliomas. Further analysis of clinical data demonstrated that higher HMGN2 expression was associated with poor prognosis in patients with glioma. The overall survival of glioma patients from the TCGA and CGGA databases was significantly shorter when HMGN2 was up-regulated (*P* < 0.0001; median cutoff; Fig. 1C). Additionally, both the CGGA mRNAseq 693 (*P* < 0.0001; median cutoff; Fig. 1C) and CGGA mRNAseq 325 (*P* < 0.0001; median cutoff; Fig. 1C) datasets showed similar trends. We analyzed HMGN2 expression levels across different molecular pathology statuses (including isocitrate dehydrogenase/IDH status, 1p 19q codeletion status, IDH & 1p 19q codeletion status,^34^ and O6-methylguanine-DNA-methyltransferase/MGMT promoter methylation status^4^^,^^35^) based on the TCGA dataset via Gliovis (http://gliovis.bioinfo.cnio.es/). We found a significant elevation in HMGN2 expression levels in the IDH wild-type group compared to the IDH mutant (mut) group (Fig. S1A). Moreover, the expression of HMGN2 demonstrates negative effects on overall patient survival across different IDH statuses (Fig. S1B). We further subdivided IDHmut and IDH wild-type into seven subtypes (Fig. S1C). The expression of G-CIMP-low was notably higher than that of the other two types in the IDH mutant group. However, no significant differences were observed between the four subtypes in the IDH wild-type group. HMGN2 expression was significantly higher in the 1p 19q non-codeletion group than in the 1p 19q codeletion group (Fig. S1D). Similarly, HMGN2 expression was higher in the IDH wild-type group than in both the IDH mut-1p 19q codeletion and IDH mut-1p 19q non-codeletion groups (Fig. S1E). Furthermore, HMGN2 expression was notably higher in the MGMT promoter-unmethylated group than in the methylated group (Fig. S1F). Based on the results of the analysis of HMGN2 expression across different molecular pathology statuses, we observed good consistency between HMGN2 expression and these classical prognostic-related pathological molecular statuses. Therefore, it can be asserted that HMGN2 expression is significantly correlated with the prognosis of gliomas.

Immunohistochemistry assays were performed to analyze the protein expression and subcellular localization of HMGN2 in a cohort of 60 paraffin-embedded archived glioma tissue samples (16 grade 2 glioma, 18 grade 3 glioma, and 26 grade 4 glioma). Among the grade 4 glioma tissues, 20 (76.9%) exhibited high positive expression of HMGN2, whereas only two (12.5%) grade 2 glioma tissues showed highly positive staining. Statistical analysis comparing the scores of HMGN2 staining across 60 archived glioma patient tissues showed that HMGN2 was widely expressed in gliomas and was significantly associated with the malignancy grade of the glioma (Fig. 1D). Additionally, as depicted in Figure 1D, the predominant locations of HMGN2 positive staining were identified within the nuclei.

### HMGN2 is related to the regulation of proliferation and cell cycle within GBM

DEGs related to HMGN2 were identified based on the TCGA database, followed by the KEGG and GO functional enrichment analyses. In the KEGG enrichment analysis, the “cell cycle” pathway was enriched with the most DEGs, indicating that “cell cycle” regulation may be involved in the impact of HMGN2 on tumor pathogenesis (*P* < 0.001; Fig. 1E, above). The top eight enriched GO terms are shown in Figure 1E. GO analysis revealed that the molecular mechanisms of these genes mainly involved the regulation of cell cycle phase transitions and cell proliferation. Biological processes for these genes that were predominantly enriched included nuclear division, organelle fission, DNA replication, and mitotic nuclear division. In terms of cellular components, the enrichment included the chromosomal region and spindle. Molecular function analysis revealed associations between tubulin and microtubule binding. Based on the prominent pathway enrichments in both KEGG and GO analyses, we conclude that HMGN2 function in glioma tumorigenesis primarily involves the cell cycle. Additionally, GSEA demonstrated a positive correlation between KEGG_CELL_CYCLE pathway genes and HMGN2 expression (*P* < 0.01; Fig. 1F). Moreover, HMGN2 was correlated with cell cycle, proliferation, and DNA damage repair function in different types of gliomas. These results were analyzed and visually represented using Cancer SEA^36^ (http://biocc.hrbmu.edu.cn/CancerSEA/home.jsp; Fig. 1G). Our analytical results based on mRNA sequencing datasets indicated an association between HMGN2 and cell cycle regulation.

### Down-regulating HMGN2 inhibits the proliferation of LN229 and U-87 MG cells

To comprehensively evaluate the expression levels of HMGN2, we conducted qPCR and Western blot analyses on five glioma cell lines, including LN229, U-87 MG, A172, U118, and U251MG. The experimental results indicated that LN229 and U-87 MG exhibited the highest expression levels, whereas U118 and A172 displayed relatively lower expression levels (Fig. S2A, B). To analyze the function of HMGN2 in GBM, LN229 and U-87 MG cells were chosen for HMGN2 knockdown using shRNAs (shHMGN2#1, shHMGN2#2, and shHMGN2#3), due to their higher HMGN2 expression levels. The knockdown efficiency was verified by qPCR and western blotting (Fig. S2C–E). We identified two sequences with good knockdown efficiencies in LN229 and U-87 MG cells (shHMGN2#1/#2 for LN229 cells and shHMGN2#2/#3 for U-87 MG cells) for subsequent experiments. Additionally, HMGN2 overexpression groups were constructed in LN229 and U-87 MG cells (Fig. S2F–H).

**Figure 2:**
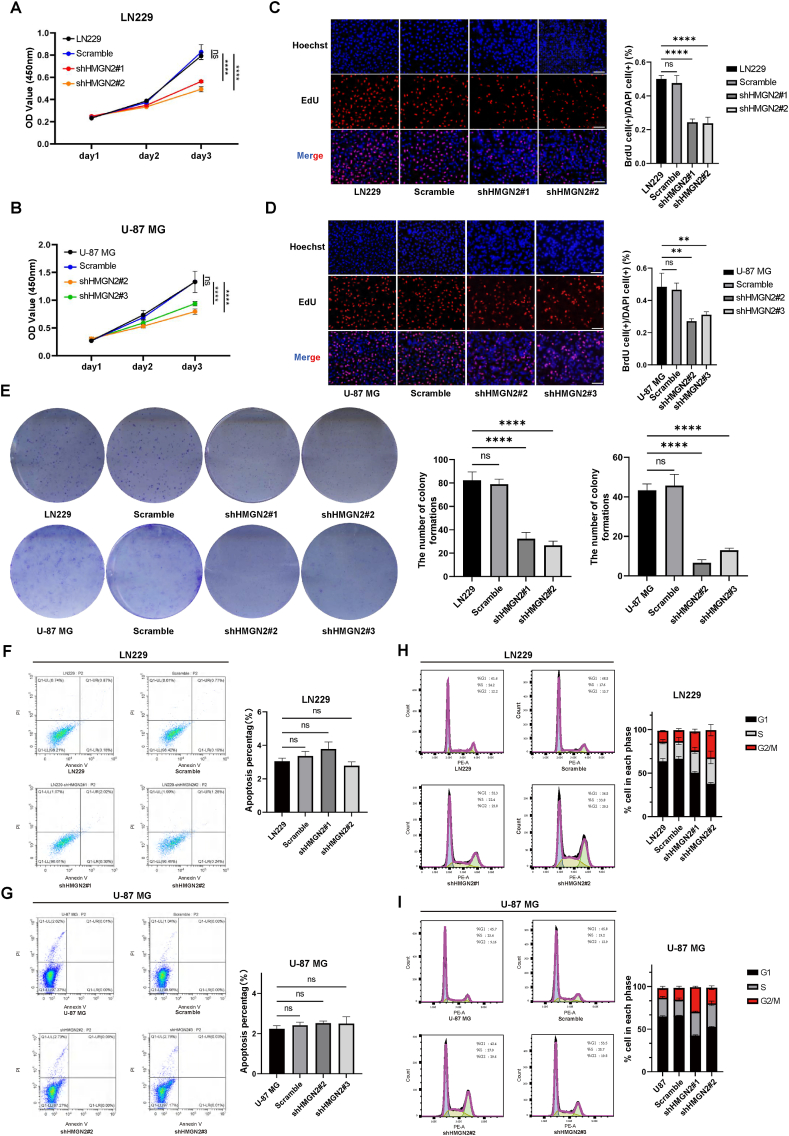
Knockdown HMGN2 inhibits the proliferation of GBM. **(A, B)** CCK-8 analysis of LN229 and U-87 MG cells in the NC, scramble, and HMGN2 knockdown groups. **(C, D)**. The EdU-positive rate of LN229 and U-87 MG cells in the NC, scramble, and HMGN2 knockdown groups. Scale bar, 60 μm. **(E)** Colony formation assay was performed in LN229 and U-87 MG cells. The NC, scramble and HMGN2 knockdown groups were plated in 6-well plates, and cells were stained with 0.05% crystal violet seven days later. **(F, G)** The apoptosis rate of LN229 and U-87 MG cells in the NC, scramble, and HMGN2 knockdown groups. The mean apoptosis rate of each group is shown on the right. **(H, I)** Analysis of cell cycle distribution of LN229 and U-87 MG in the NC, scramble, and HMGN2 knockdown groups. The mean percentages of the population in each phase are shown on the right. All data were expressed as mean ± standard deviation. Student's *t*-test was performed to analyzed significance; ∗*P* < 0.05, ∗∗*P* < 0.01, ∗∗∗*P* < 0.001, and ∗∗∗∗*P* < 0.0001.

**Figure 3:**
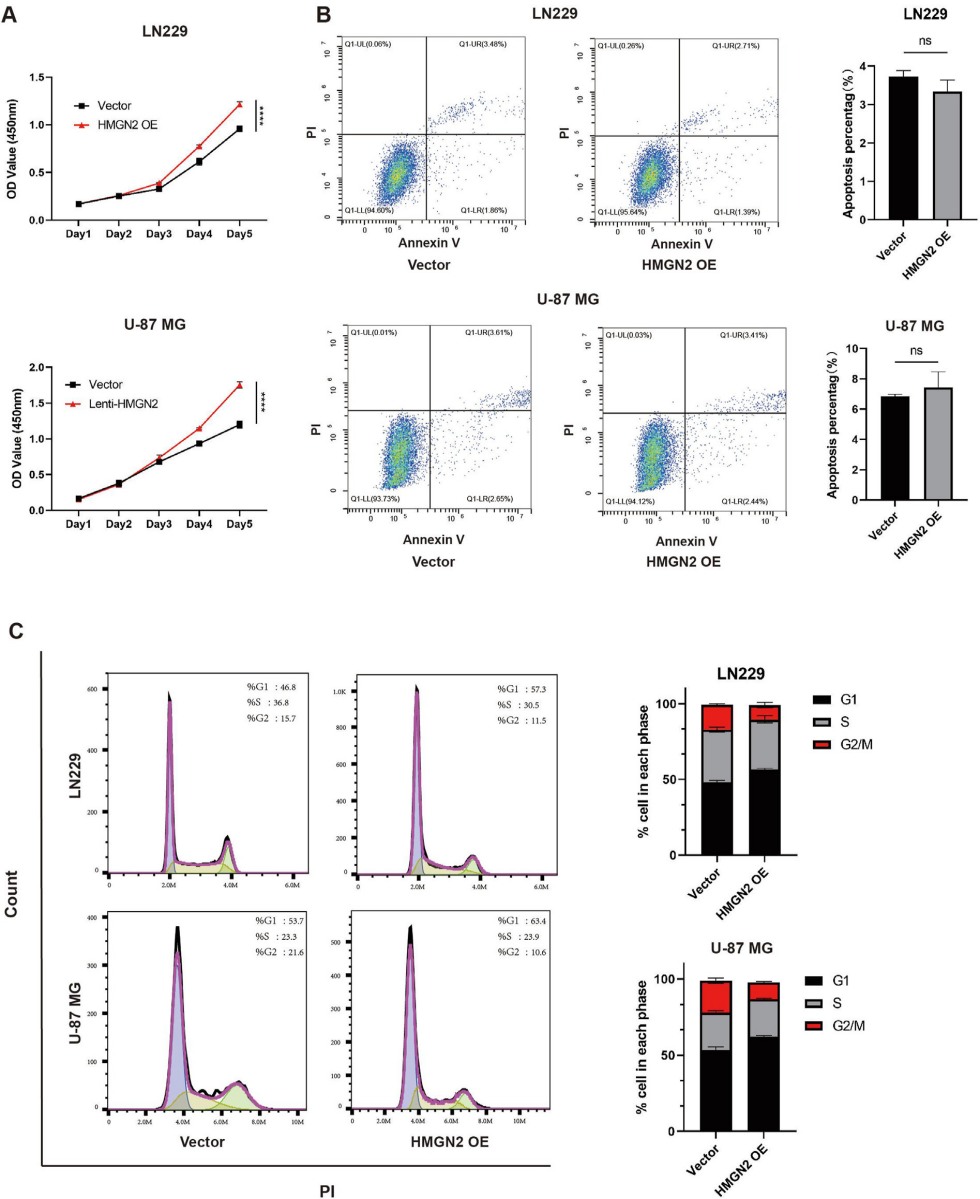
Overexpression HMGN2 promotes the proliferation of glioblastoma multiforme. **(A)** CCK-8 analysis of LN229 and U-87 MG cells in the VC and HMGN2 OE groups. **(B)** The apoptosis rate of LN229 and U-87 MG cells in the VC and HMGN2 OE groups. The mean apoptosis rate of each group is shown on the right. **(C)** Analysis of cell cycle distribution of LN229 and U-87 MG in the VC and HMGN2 OE groups. The mean percentages of the population in each phase are shown on the right. All data were expressed as mean ± standard deviation. Student's *t*-test was performed to analyzed significance; ∗*P* < 0.05, ∗∗*P* < 0.01, ∗∗∗*P* < 0.001, and ∗∗∗∗*P* < 0.0001.

To evaluate the impact of HMGN2 on the proliferative capacity of glioma cells, we employed CCK8 and EdU assays to assess cell viability and DNA synthesis, respectively. The results showed that the knockdown of HMGN2 in LN229 and U-87 MG cells significantly inhibited cell viability and proliferation compared with the NC and scramble groups on the third day (Fig. 2A, B). Additionally, the proportion of EdU-positive cells sharply decreased in the HMGN2 knockdown group (Fig. 2C, D), indicating that a lower proportion of cells entered the S phase of the DNA replication cycle. Moreover, consistent results were observed in the colony formation assay. HMGN2 knockdown in GBM cells resulted in diminished proliferative capacity with fewer colony counts and a significantly smaller average clone size (Fig. 2E). To exclude the possibility that the inhibitory effect of HMGN2 was attributed to increased apoptosis, we assessed apoptotic rates using annexin V staining and flow cytometry (Fig. 2F, G). We further assessed the cell cycle phase distribution using flow cytometry. The results showed different degrees of G1 phase reduction and G2/M phase increase in LN229 and U-87 MG cells after HMGN2 knockdown (Fig. 2H, I; Fig. S2I). A similar effect of HMGN2 on cell viability, apoptosis, and cell cycle distribution was observed in the HMGN2 overexpression group in LN229 and U-87 MG cells (Fig. 3A–C; Fig. S2J), supporting the notion that HMGN2 exerts a pro-proliferative effect on GBM cells.

To confirm our findings, we used glioma stem-like cells TS543 to establish shHMGN2 cell lines (Fig. S3A) and conducted CCK8 assays (Fig. S3B) and cell cycle analysis (Fig. S3C). These results are consistent with those observed in the LN229 and U-87 MG knockdown groups. These results indicate that HMGN2 may promote the proliferative capacity of glioma cells by regulating the cell cycle, which is consistent with the predictions from bioinformatics analysis.

### HMGN2 knockdown inhibits tumor growth *in vivo*

**Figure 4:**
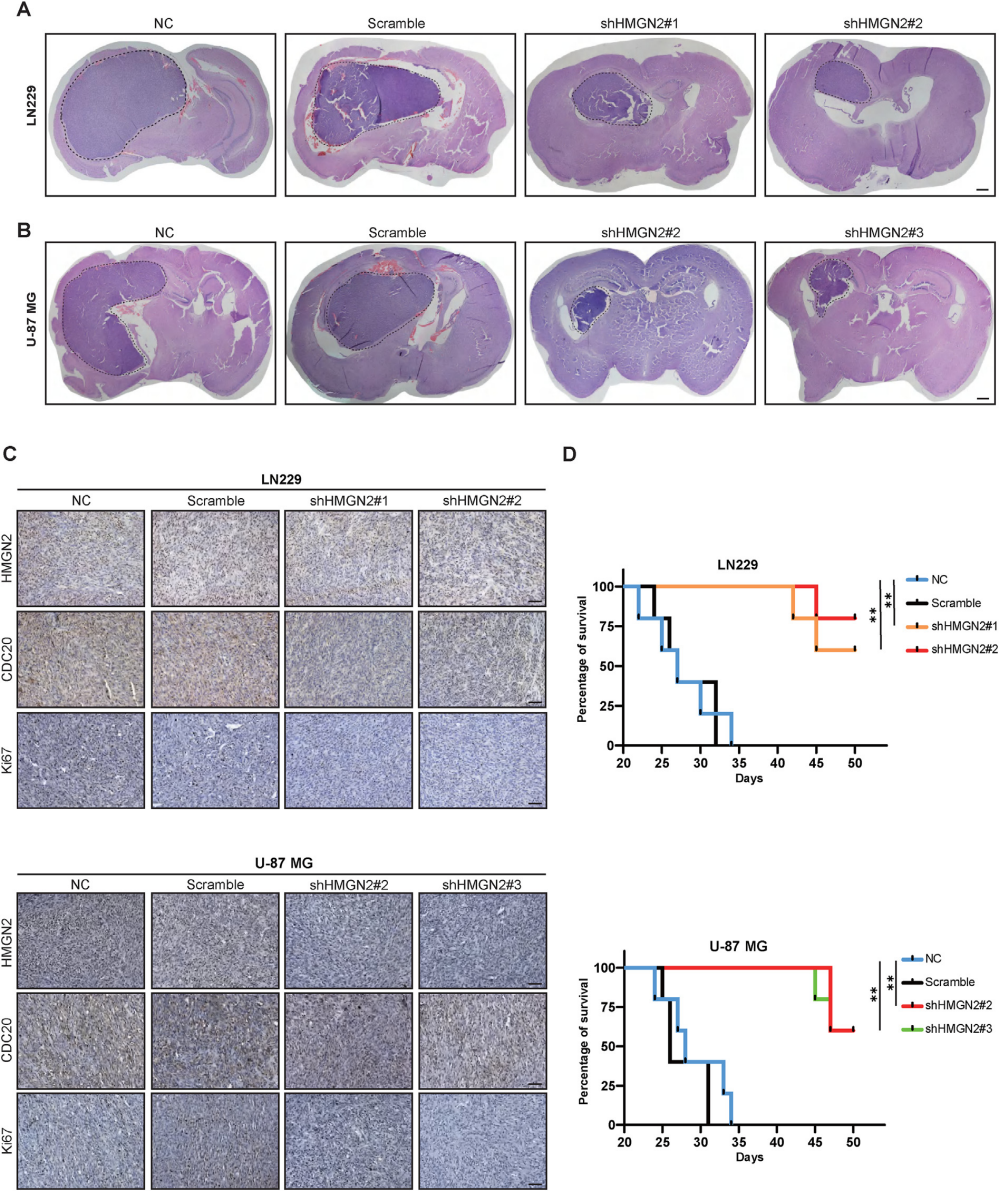
HMGN2 promotes tumor progression *in vivo*. **(A, B)** Establishing intracranial xenografts after knockdown of HMGN2 and the NC and scramble groups in LN229 and U-87 MG cells. Scale bar, 1 mm. **(C)** Detection of the expression levels of HMGN2, CDC20, and Ki67 in cell line-derived xenograft (CDX) tumors by immunohistochemical staining. Scale bar, 50 μm. **(D)** Mouse survival times were recorded after the establishment of intracranial xenografts and for each group. *n* = 5; ∗*P* < 0.05, ∗∗*P* < 0.01, ∗∗∗*P* < 0.001, and ∗∗∗∗*P* < 0.0001.

To investigate the inhibitory effect of HMGN2 knockdown on GBM proliferation *in vivo*, the HMGN2 knockdown and control groups were injected with NOD/SCID mice to generate intracranial xenograft models. As expected, HMGN2 knockdown significantly reduced tumor size compared with the NC group and the scrambled group (Fig. 4A, B). In the meantime, immunohistochemical staining demonstrated a marked down-regulation of HMGN2 and Ki67 expression in the HMGN2 knockdown group (Fig. 4C). Additionally, the survival time of mice was prolonged in the HMGN2 knockdown group (Fig. 4D). Overall, HMGN2 knockdown caused a significant change in tumor size and survival time *in vivo*.

### HMGN2 promotes GBM proliferation by regulating CDC20 expression

**Figure 5:**
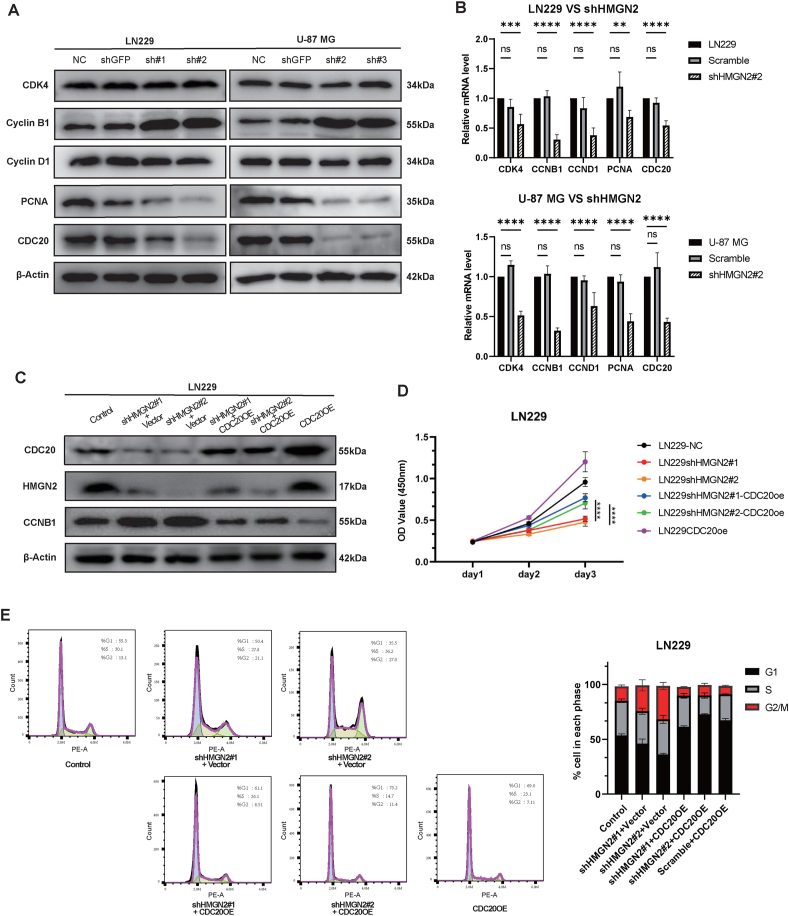
HMGN2 promotes glioblastoma multiforme proliferation through CDC20 regulating cell cycle. **(A)** The levels of cell cycle-related proteins were examined by Western blot in LN229 and U-87 MG cells of the NC, scramble, and HMGN2 knockdown groups. **(B)** The mRNA levels of cell cycle-related genes were examined by qPCR in LN229 and U-87 MG cells of the NC, scramble, and HMGN2 knockdown groups. ∗*P* < 0.05, ∗∗*P* < 0.01, ∗∗∗*P* < 0.001, and ∗∗∗∗*P* < 0.0001. **(C)** CDC20 and CCNB1 expression levels were detected by Western blot in the CDC20 rescue assay. **(D)** The proliferation ability of the CDC20 rescue assay in LN229 cells was evaluated by CCK-8 assay. **(E)** Cell cycle distribution in the CDC20 rescue assay was detected by flow cytometry. The mean percentages of the population in each phase are shown on the right. All data were expressed as mean ± standard deviation. Student's *t*-test was performed to analyzed significance; ∗*P* < 0.05, ∗∗*P* < 0.01, ∗∗∗*P* < 0.001, and ∗∗∗∗*P* < 0.0001.

These results suggest that HMGN2 is essential for cell cycle regulation. To verify this, we performed transcriptome sequencing of the LN229 shHMGN2#2 and scramble groups. Subsequently, differential gene analysis was performed on the sequencing results. The DEGs were enriched by KEGG and GO (Fig. S4A and Table S2), and genes enriched in functions associated with the cell cycle were extracted. In parallel, we performed a similar analysis on public transcriptome data from the TCGA and CGGA databases, extracting differential genes enriched in cell cycle-related functions (Table S2). We identified 12 cell cycle-related genes that were differentially expressed in the HMGN2 high versus low groups in the TCGA database, and also in the HMGN2 knockdown versus control groups in our mRNA sequencing data (CDK1, CDK2, CDK4, CDK6, CCNB1, CCNB2, CCNC, CCND1, CCNE1, CCNE2, PCNA, and CDC20). Subsequently, we measured the mRNA levels in the knockdown and overexpression LN229 and U-87 MG cell lines (Fig. 5B; Fig. S4B). Among these genes, only five were significantly regulated at the transcriptional level. Western blotting results showed that only CCNB1 (cyclin B1), PCNA (proliferating cell nuclear antigen), and CDC20 were notably associated with HMGN2 changes in GBM (Fig. 5A; Fig. S4C, D).

Among them, CDC20 consistently showed the most significant alterations at both mRNA and protein levels. HMGN2 knockdown led to a decrease in the transcription level of CDC20 mRNA, and its protein level also showed a substantial decreasing trend. A consistent regulatory mechanism was also shown in the HMGN2 overexpression group. However, CCNB1 showed an opposite trend at both the mRNA and protein levels. This discrepancy was explained by the reduction in CDC20, leading to a decrease in the formation of APC-CDC20 complexes in the G2/M phase and causing the accumulation of CCNB1. Consequently, despite the decline in the transcript level of CCNB1 upon HMGN2 knockdown, the protein level displayed an increasing trend owing to the reduced degradation of CCNB1 (Fig. 5A; Fig. S4C). This regulatory mechanism was also observed in the HMGN2 overexpression group (Fig. S4D), which offered a reasonable explanation for the observed trends.

To confirm the role of CDC20 in the HMGN2-induced regulation of the GBM cell cycle, we conducted rescue experiments by transfecting CDC20 overexpression lentiviruses into the LN229 shHMGN2 group. We observed a significant restoration of CDC20 protein levels in the shHMGN2#1-CDC20OE and shHMGN2#2-CDC20OE groups (Fig. 5C). CCK8 assay results showed that the cell proliferation capacity was increased relative to the shHMGN2#1 and shHMGN#2 groups (*P* < 0.01; Fig. 5D). Additionally, cell cycle analysis showed a noteworthy decrease in the percentage of cells blocked in the G2/M phase (Fig. 5E; Fig. S4E). The rescue experiment further showed that HMGN2 knockdown regulated the cell cycle by decreasing the level of CDC20, consequently attenuating the proliferative ability of glioma cells. Simultaneously, we measured the protein level of CCNB1 in the CDC20 recovery experiment. Similar to our previous speculation (Fig. 5C), overexpression of CDC20 increased the level of the APC-CDC20 complex, which accelerated the degradation of CCNB1 and caused a considerable decline in the protein level compared with that in the shHMGN2 group. The protein level decreased even more noticeably when only CDC20 was overexpressed without HMGN2 knockdown.

### The regulatory mechanism of HMGN2 on CDC20

**Figure 6:**
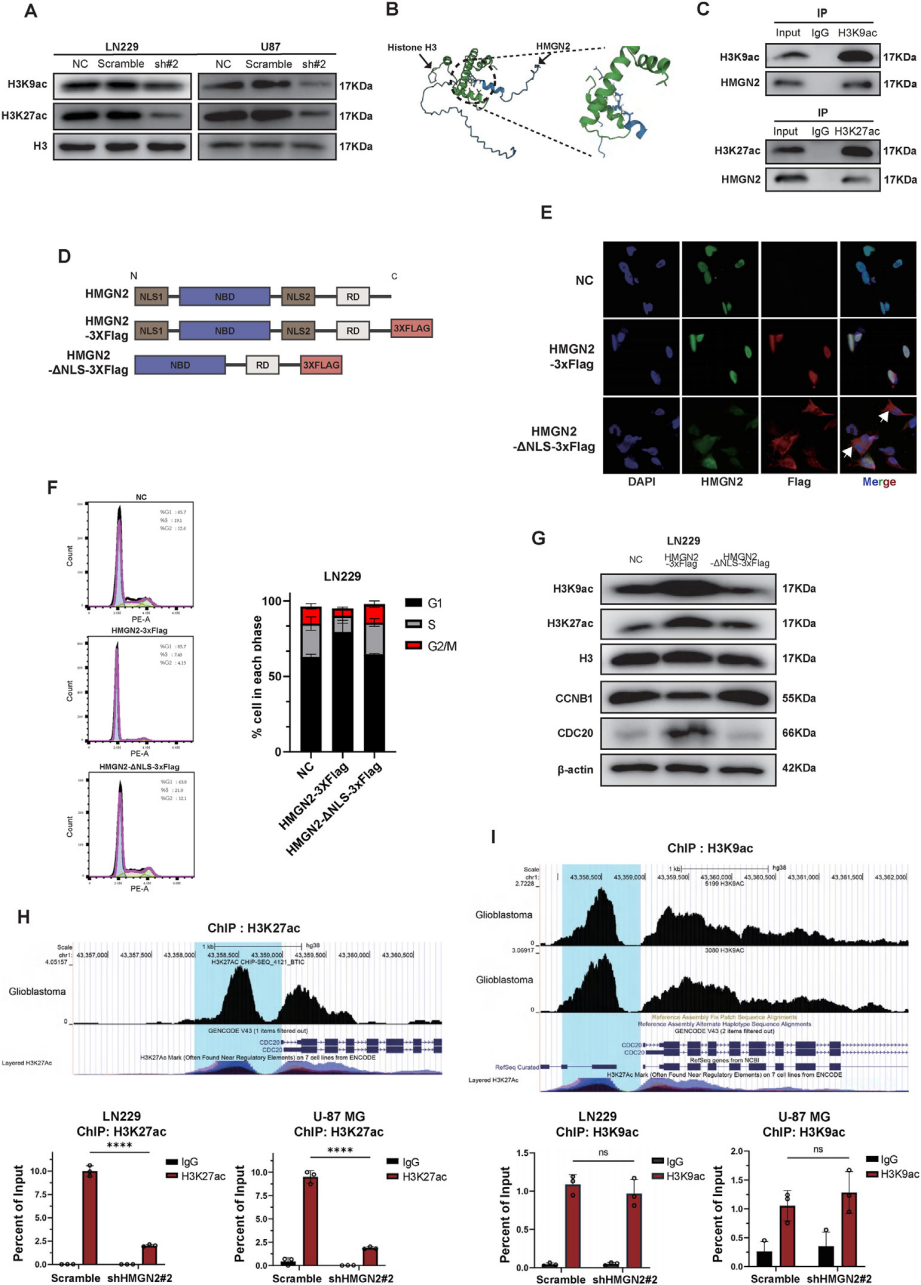
The mechanism of HMGN2 regulating CDC20. **(A)** The levels of H3K9ac and H3K27ac in LN229 and U-87 MG cells of the NC, scramble, and HMGN2 knockdown groups were detected by Western blot. **(B)** The visual analysis of HMGN2 binding with H3. **(C)** HMGN2 binding with H3K9ac and H3K27ac were detected by co-immunoprecipitation assay in LN229 cells. **(D)** The schematic structure of HMGN2 and the truncated HMGN2 used in this study. HMGN2 contains four major domains: two nuclear localization signal domains (NLS1, residues 1–16; NLS2, residues 47–67), a nucleosome binding domain (NBD, residues 17–46), and a regulatory domain (RD, residues 68–89). **(E)** The immunofluorescence experiment was performed to locate HMGN2 and truncated HMGN2. **(F)** The cell cycle distribution was detected by flow cytometry in the NC, HMGN2-3XFlag, and HMGN2-ΔNLS-3XFlag groups. The mean percentages of the population in each phase are shown on the right. **(G)** The levels of H3K9ac, H3K27ac, CCNB1, and CDC20 in the NC, HMGN2-3XFlag, and HMGN2-ΔNLS-3XFlag groups were detected by Western blot. **(H, I)** The analysis from CistromeDB (https://cistrome.org) for H3K27ac and H3K9ac binding peaks in the promoter region of CDC20 in glioblastoma multiforme tissues on the top. Chromatin immunoprecipitation analysis of H3K9ac and H3K27ac on the CDC20 promoter in LN229 and U-87 MG cells with or without HMGN2 knockdown (2% input of each group was pulled down and applied to qPCR). All data were expressed as mean ± standard deviation. Student's *t*-test was performed to analyzed significance; ∗*P* < 0.05, ∗∗*P* < 0.01, ∗∗∗*P* < 0.001, and ∗∗∗∗*P* < 0.0001.

Previous studies on the role of HMGN2 in downstream regulation have shown that HMGN2 mainly participates in the transcriptional regulation of downstream molecules. It interacts predominantly with histones, promoter regions, and transcription factors within the nucleus.^10^, ^11^, ^12^ Previous studies have reported that HMGN2 can modulate the acetylation levels of histones. Western blot results also showed that the acetylation levels of H3K27 and H3K9 were significantly decreased in LN229 and U-87 MG cells after HMGN2 knockdown compared with the NC and scramble groups (Fig. 6A). Visual analysis revealed interactions between the amino acids in the NBD of HMGN2 and H3, leading to stable binding (Fig. 6B). Moreover, the binding of H3K27ac and H3K9ac to HMGN2 in LN229 was verified by co-immunoprecipitation experiments (Fig. 6C). The acetylation levels of H3K27 and H3K9 serve as markers of chromatin accessibility and transcriptional activity in the relevant regions. We speculate that HMGN2 affects transcriptional activity by binding to histones in glioma cells, thereby increasing their acetylation levels and resulting in increased chromatin transcriptional activity.

We deleted the nuclear localization signal (NLS) of HMGN2 based on a previously reported domain distribution of HMGN2^37^ and combined it with website predictions (https://smart.embl.de/). This was performed to clarify whether the regulatory mode relied on transcriptional regulation after nuclear import (Fig. 6D). Cellular immunofluorescence experiments clearly showed that, in the NC group, the endogenous HMGN2 fluorescence signal, labeled with an anti-HMGN2 antibody, was localized in the nucleus. The fluorescence signal highly overlapped with the nuclear signal of DAPI (Fig. 6E) and was consistent with the immunohistochemistry results (Fig. 1D). In the HMGN2-3XFlag group, the HMGN2 and Flag signals overlapped within the DAPI-stained nuclear region. Conversely, in the HMGN2-ΔNLS-3XFlag group, it was apparent that NLS's deletion did not affect the labeling of truncated HMGN2 by anti-HMGN2 antibody. However, it can be observed that the area of anti-HMGN2 antibody labeling in the HMGN2-ΔNLS-3XFlag group surpasses the confines of the DAPI nuclear region. As shown by the white arrows, the anti-FLAG antibody-labeled truncated HMGN2 is localized outside the nucleus, indicating that full-length HMGN2 is localized in the nucleus, and the deletion of its NLS can restrict some or all the truncated proteins from localizing to the cytoplasm. Upon comparing the NC group with the HMGN2-3XFlag group, we found that truncated HMGN2 lost its regulatory effect on the cell cycle when the NLS was removed, whereas the functional domains were retained (Fig. 6F; Fig. S5F). The influence on the expression of CCNB1 and CDC20 proteins, as well as the acetylation levels of H3K9 and H3K27 proteins, disappeared when HMGN2 could not be imported into the nucleus (Fig. 6G). Therefore, we believe that the regulatory mechanism of HMGN2 depends on its transcriptional levels after nuclear transport.

Through analysis of public ChIP-sequencing data from CistromeDB (https://cistrome.org), we identified the binding peaks of H3K27ac and H3K9ac in the promoter region of CDC20 (Fig. 6H, I; Fig. S5A, B). The enrichment abundance in tumors was significantly higher than that in normal brain tissue. Their transcriptional activity may be regulated by two acetylation modifications. To investigate whether HMGN2 regulates CDC20 transcription by modulating the acetylation levels of these two residues, we conducted ChIP-qPCR experiments to assess histone acetylation levels in the CDC20 promoter region in both the control and shHMGN2#2 groups. The results of ChIP-qPCR experiments on H3K27ac and H3K9ac showed that the amount of H3K27ac in the CDC20 promoter region decreased significantly when HMGN2 was knocked down (Fig. 6H), whereas the change in H3K9ac was not statistically significant (Fig. 6I). In summary, our results show that HMGN2 is involved in the transcriptional regulation of CDC20 by modulating the acetylation level of H3K27 in the CDC20 promoter region, thereby affecting the cell cycle distribution and proliferative ability of gliomas.

### CDC20 is up-regulated in glioma and correlates with patient prognosis

**Figure 7:**
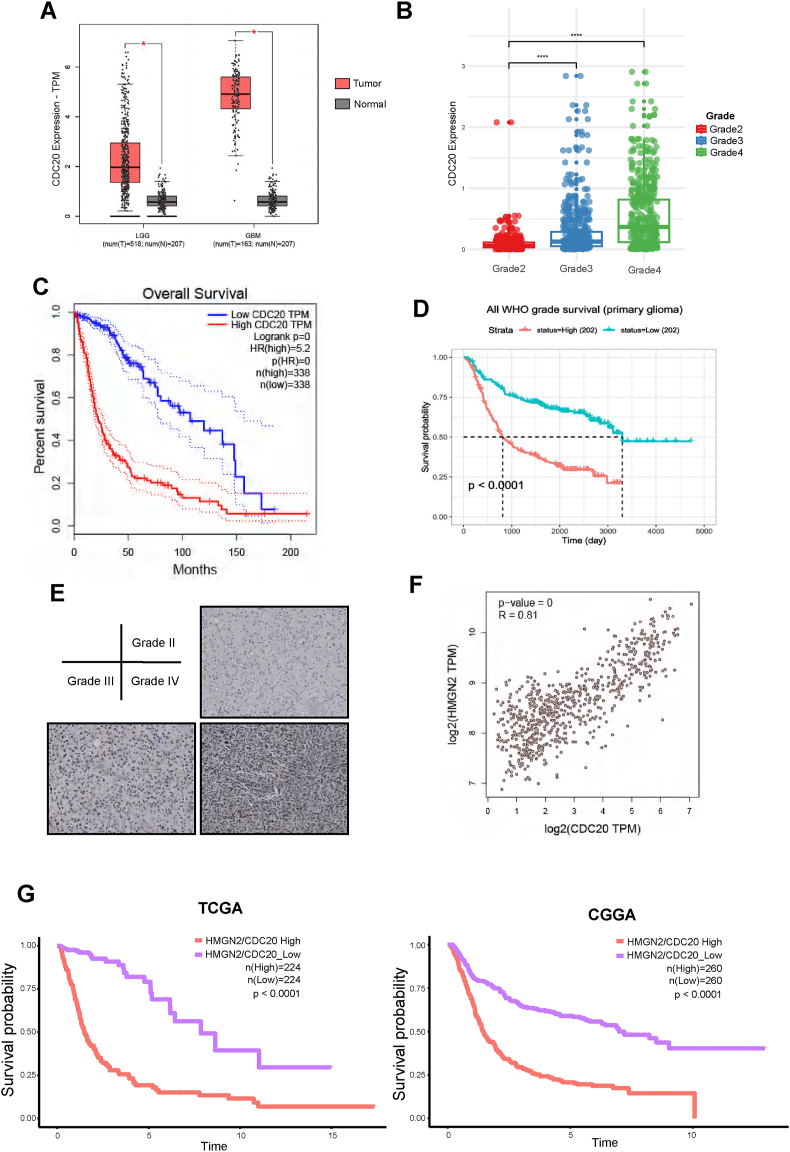
CDC20 was up-regulated in human glioma tissues and correlated with the prognosis of glioma patients. **(A)** The expression level of the HMGN2 (mRNA sequencing) in low-grade gliomas (LGG) and glioblastoma multiforme (GBM) from GEPIA. **(B)** The expression level of the HMGN2 in different grades of glioma from CGGA-693. **(C, D)** The prognostic significance of CDC20 in glioma via TCGA and CGGA-693 databases. **(E)** Immunohistochemical staining was performed to evaluate CDC20 expression in glioma tissue samples. Scale bar, 50 μm. **(F)** Pearson correlation coefficient plots between HMGN2 and CDC20. **(G)** The overall survival time plot of patients in the high HMGN2/CDC20 expression group versus those in the low HMGN2/CDC20 expression group. All data were expressed as mean ± standard deviation. Student's *t*-test was performed to analyze significance; ∗*P* < 0.05, ∗∗*P* < 0.01, ∗∗∗*P* < 0.001, and ∗∗∗∗*P* < 0.0001.

Taken together, these results demonstrate that HMGN2 promotes GBM proliferation by up-regulating CDC20 expression. The CDC20 expression plot from GEPIA and CGGA showed that CDC20 expression was up-regulated in GBM compared with that in low-grade gliomas (Fig. 7A, B). Therefore, we drew survival curves for glioma patients in GEPIA and CGGA. The disease-free survival curve indicated that CDC20 expression was negatively correlated with the survival time of glioma patients (Fig. 7C, D). Moreover, we collected glioma tissues for immunohistochemistry assay to detect CDC20 expression. The CDC20 expression in the human glioma tissues was also up-regulated parallel to the tumor grade (Fig. 7E), consistent with mRNA sequencing data analysis. Taken together, these results demonstrated that HMGN2 and CDC20 expression were up-regulated in human glioma tissues, and we observed a clear correlation between CDC20 and HMGN2 expression (Fig. 7F). Additionally, the overall survival of patients in the high HMGN2/CDC20 expression group was significantly different from that of patients in the low HMGN2/CDC20 expression group (Fig. 7G).

## Discussion

Although efforts have been made to improve the treatment of gliomas, targeted agents and immunotherapies targeting the genetic or biological hallmarks of gliomas have not yielded satisfactory results.^3^, ^4^, ^5^, ^6^, ^7^ An increasing number of studies have confirmed that epigenetic alterations, including DNA methylation, histone post-translational modifications, and chromatin organization, play critical roles in tumor proliferation.^38^, ^39^, ^40^ Thus, epigenetic alterations represent potential breakthroughs in glioma treatment.

The importance of HMGN2 in embryonic development,^41^ lineage characterization and maintenance,^42^ controlling of cell type-specific gene expression, and tumor progression and inhibition^43^^,^^44^ has drawn our attention. The current study suggests that HMGN2 affects chromatin structure by competing for histone-binding sites and regulating histone post-translational modifications, thereby regulating gene expression. However, whether it promotes or inhibits gliomas and the underlying molecular mechanisms remain unclear. In this study, we demonstrate that HMGN2 promotes the proliferation of glioma cells *in vitro* and *in vivo* and that glioma cells experience a slowdown in proliferation when HMGN2 is knocked down. The results of the bioinformatics analysis were consistent with the flow cycle results, which indicated that HMGN2 could regulate proliferation in gliomas through the regulation of the G2/M phase of the cell cycle.

Unbridled proliferation and abnormal activity of the cell cycle are vital for cancer cell progress and represent a driving force of tumorigenesis.^45^ CDC20 is a regulator of the cell cycle as it controls the correct segregation of chromosomes during mitosis.^46^ Several researchers have reported that the overexpression of CDC20 is associated with prognosis in bladder cancer,^47^ hepatocellular carcinoma,^28^ and triple-negative breast cancer^48^ and exerts different effects on different tumor types. For example, high CDC20 expression increases tumor cell growth and migration in triple-negative breast cancer. In hepatocellular carcinoma and bladder cancer, CDC20 is implicated in radio-resistance and proliferation. Screening for DEGs revealed that CDC20 expression was significantly down-regulated when HMGN2 was knocked down. Additionally, our rescue experiments showed that restoration of CDC20 expression reversed the inhibitory effect of HMGN2 knockdown on glioma proliferation and reduced the proportion of cells in the G2/M phase. In conclusion, alterations in the cell cycle induced by CDC20 play an important role in glioma cell proliferation resulting from HMGN2 knockdown.

We also investigated the mechanism underlying HMGN2's regulating CDC20 expression. By binding to histone-binding sites and regulating histone post-translational modifications, HMGN2 can restrict chromatin concentration and control gene expression. In breast cancer, HMGN2 specifically promotes STAT5 accessibility to the promoter DNA by facilitating the dissociation of the linker histone H1. This increases Stat5a-mediated transcription and elevates the protein expression of breast cancer proliferative genes, such as CISH, CEBPb (CCAAT/enhancer binding protein β), and cyclin D1, which promote the proliferation of breast cancer.^13^^,^^14^ Moreover, the above regulatory mechanism does not rely on a specific DNA sequence. It is thought to be attracted by the epigenetic marks of active chromatin.^12^

In-depth investigations have shown that HMGN2 knockdown not only regulates DEGs, such as CDC20, but also decreases the levels of acetylation of H3K9 and H3K27. Studies have shown that the binding of HMGN2 to histones can regulate the affinity of histone acetylase and histone deacetylase to histones, thereby increasing the equilibrium point of the degree of acetylation of intercellular histones. The impact of HMGN2 on histones is selective, affecting specific histones rather than every histone. Currently, HMGN2 is believed to be more likely to be attracted to acetylated histones. Genes with higher chromatin accessibility are more likely to be influenced by HMGN2 to maintain and enhance chromatin activity, which has a positive feedback effect on the epigenetic regulation of genes.

Through the analysis of publicly available ChIP-sequencing data, we identified enrichment peaks of H3K27ac and H3K9ac by mapping to the promoter region of CDC20. We also observed that the promoter enrichment of H3K27ac and H3K9ac at CDC20 was more significant in glioblastoma and glioma cell lines than in healthy brain tissue. However, the results of our ChIP-qPCR experiments showed that only H3K27ac was involved in the regulation of CDC20 by HMGN2. Additionally, the PCNA promoter region showed clear enrichment peaks (Fig. S5C, D). ChIP-qPCR confirmed that acetylation of H3K27 and H3K9 was involved in the regulation of PCNA by HMGN2 (Fig. S5E). This could be one of the mechanisms how HMGN2 downstream genes are epigenetically regulated, affecting gene transcriptional activity and altering gene expression.

**Figure 8:**
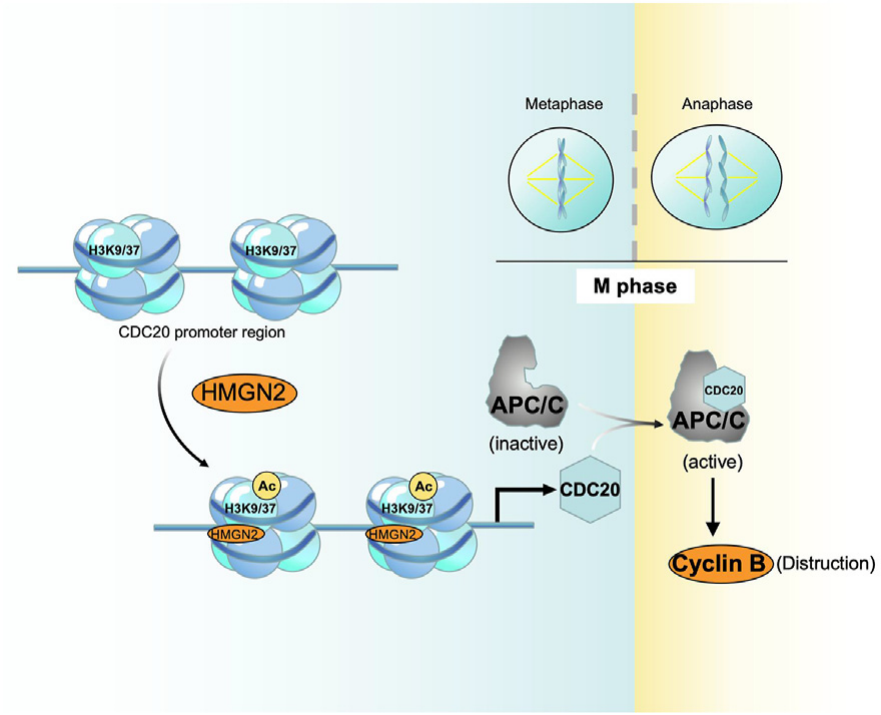
The model map of this research.

In this study, we examined the activation marker of histone acetylation and demonstrated that HMGN2 downstream regulation is based on the epigenetic regulation of histone post-translational modifications. This regulation enhances chromatin accessibility in the promoter regions of CDC20 and PCNA. During transcription, the recruitment of TFIID to chromatin can be facilitated by histone acetylation,^49^ promoting the assembly of transcription-initiating complexes, thereby promoting the expression of downstream genes (Fig. 8). However, whether the binding of specific transcription factors or activators is enhanced during transcription is worth further exploration, and we intend to investigate this mechanism in future studies. Finally, we conducted a clinical analysis using the CGGA and TCGA datasets and our samples and discovered that CDC20 was overexpressed in high-grade gliomas versus low-grade gliomas and normal brain tissue. Additionally, gliomas with high levels of CDC20 are associated with a poor prognosis. HMGN2 and CDC20 expression levels are positively correlated in glioma tissues. In summary, these results indicated that HMGN2 and CDC20 are crucial for glioma progression and may serve as potential biomarkers for predicting the prognosis of patients with glioma.

Overall, our study shows that HMGN2 enhances glioma cell proliferation both *in vitro* and *in vivo*. Additionally, HMGN2 positively regulated acetylation of the CDC20 promoter region. This enhances chromatin accessibility, consequently promoting glioma cell proliferation. Finally, we identified HMGN2 and CDC20 as potential biomarkers and therapeutic targets for the treatment of glioma.

## Ethics declaration

All animal experiments were conducted in accordance with the regulations of the Animal Protection and Use Committee of Southwest University and the Guide for the Protection and Use of Laboratory Animals (Ministry of Science and Technology of China, 2006). Clinical tissue samples were obtained with approval from the Ethics Committee of The First Affiliated Hospital of Chongqing Medical University (No. K2023-551). Informed consent was waived for this study, as it used medical records and biological specimens obtained during previous clinical diagnoses and treatments.

## Data availability

All data generated and analyzed for this study are included in this manuscript.

## CRediT authorship contribution statement

**Jiacheng Zhong:** Conceptualization, Data curation, Formal analysis. **Shuang Shi:** Formal analysis, Investigation, Methodology, Validation. **Wen Peng:** Resources, Supervision, Writing – original draft. **Hongjuan Cui:** Resources, Supervision, Validation, Writing – original draft. **Xiaochuan Sun:** Supervision, Validation, Writing – original draft.

## Conflict of interests

The authors declare no commercial or financial ties that might be seen as possible conflicts of interest during the research.

## Appendix A Supplementary data

Multimedia component 1 Multimedia component 1

**Figure S1.** HMGN2 expression across different molecular pathological statuses. **(A, B)** HMGN2 expression levels in different IDH (isocitrate dehydrogenase) statuses and overall patient survival time across different IDH statuses. **(C)** Analysis of HMGN2 expression in seven subtypes of glioma based on the TCGA dataset (G-CIMP-low, G-CIMP-high, Codel, Classic-like, Mesenchymal-like, LGm6-GBM, PA-like). **(D)** HMGN2 expression levels in different 1p 19q codeletion statuses based on the TCGA dataset. **(E)** HMGN2 expression levels in different IDH and 1p 19q codeletion statuses based on the TCGA dataset. **(F)** HMGN2 expression levels in different MGMT (O6-methylguanine-DNA-methyltransferase) promoter methylation statuses based on the TCGA dataset. ∗*P* < 0.05, ∗∗*P* < 0.01, ∗∗∗*P* < 0.001, and ∗∗∗∗*P* < 0.0001.

**Figure S2.** HMGN2 expression in glioblastoma multiforme cell lines and knockdown or overexpression in glioblastoma multiforme. **(A, B)** HMGN2 expression was measured by qPCR and western blotting in five glioblastoma multiforme cell lines (LN229, U-87 MG, A172, U118, and U251). **(C–H)** HMGN2 expression was detected after HMGN2 knockdown or overexpression using reverse-transcription PCR analysis and western blotting. **(I)** Analysis of cell cycle distribution of LN229 and U-87 MG cells in the NC, scramble, and HMGN2 knockdown groups. The mean percentages of the population in the G2/M phase were plotted. **(J)** Analysis of cell cycle distribution of LN229 and U-87 MG cells in the VC and HMGN2 OE groups. The mean percentages of the population in the G2/M phase were plotted. All data were expressed as mean ± standard deviation. One-way ANOVA and student's *t*-test were performed to analyze significance; ∗*P* < 0.05, ∗∗*P* < 0.01, ∗∗∗*P* < 0.001, and ∗∗∗∗*P* < 0.0001.

**Figure S3.** HMGN2 knockdown inhibits the glioma stem-like cell proliferation. **(A)** HMGN2 expression in TS543 glioma stem-like cells was measured by western blotting. **(B)** CCK-8 analysis of TS543 cells in the NC, scramble, and HMGN2 knockdown groups. **(C)** Analysis of cell cycle distribution of TS543 cells in the NC, scramble, and HMGN2 knockdown groups. The mean percentages of the population in the G2/M phase were plotted. One-way ANOVA was performed to analyze significance; ∗*P* < 0.05, ∗∗*P* < 0.01, ∗∗∗*P* < 0.001, and ∗∗∗∗*P* < 0.0001.

**Figure S4.** HMGN2 regulated cell cycle-related proteins. **(A)** The KEGG and GO enrichment analyses based on the differentially expressed genes from RNA sequencing of LN229 shHMGN2#2 versus scramble groups. **(B)** Left: The mRNA levels of 12 cell cycle-related genes were examined by qPCR in LN229 and U-87 MG cells of the NC, scramble, and HMGN2 knockdown groups; Right: The mRNA levels of cell cycle-related genes were examined by qPCR in LN229 and U-87 MG cells of the NC, Vector, and HMGN2 OE groups. **(C)** Semi-quantitative analysis of the cell cycle-related proteins by GraphPad. **(D)** The levels of cell cycle-related proteins were examined by western blotting in LN229 and U-87 MG cells of the NC, Vector, and HMGN2 OE groups. **(E)** Analysis of cell cycle distribution of LN229 cells in rescue experiments. The mean percentages of the population in the G2/M phase were plotted. One-way ANOVA was performed to analyze significance; ∗*P* < 0.05, ∗∗*P* < 0.01, ∗∗∗*P* < 0.001, and ∗∗∗∗*P* < 0.0001.

**Figure S5.** H3K27ac and H3K9ac binding peaks in the CDC20 and PCNA promoters. **(A–D)** The analysis from CistromeDB (https://cistrome.org) for H3K27ac and H3K9ac binding peaks in the promoter regions of CDC20 and PCNA in different tissues and cells. **(E)** Chromatin immunoprecipitation analysis of H3K9ac and H3K27ac on the PCNA promoter in LN229 and U-87 MG cells with or without HMGN2 knockdown (2% of the input from each group was pulled down and subjected to qPCR). **(F)** The cell cycle distribution was detected by flow cytometry in the NC, HMGN2-3XFlag, and HMGN2-ΔNLS-3XFlag groups. The mean percentages of the population in the G2/M phase were plotted. All data were expressed as mean ± standard deviation. One-way ANOVA and student's *t*-test were performed to analyze significance; ∗*P* < 0.05, ∗∗*P* < 0.01, ∗∗∗*P* < 0.001, and ∗∗∗∗*P* < 0.0001.
