# Supplementary material for: HMGN2 accelerates the proliferation and cell cycle progression of glioblastoma by regulating CDC20 expression
Source: Genes Dis. 2024 Sep 12;12(3):101433. doi: 10.1016/j.gendis.2024.101433 (PMC11907455; doi:10.1016/j.gendis.2024.101433)
Supplement: Multimedia component 1 [file mmc1.docx]

**Figure S1** HMGN2 expression across different molecular pathological statuses. **(A, B)** HMGN2 expression levels in different IDH (isocitrate dehydrogenase) statuses and overall patient survival time across different IDH statuses. **(C)** Analysis of HMGN2 expression in seven subtypes of glioma based on the TCGA dataset (G-CIMP-low, G-CIMP-high, Codel, Classic-like, Mesenchymal-like, LGm6-GBM, PA-like). **(D)** HMGN2 expression levels in different 1p 19q codeletion statuses based on the TCGA dataset. **(E)** HMGN2 expression levels in different IDH and 1p 19q codeletion statuses based on the TCGA dataset. **(F)** HMGN2 expression levels in different MGMT (O6-methylguanine-DNA-methyltransferase) promoter methylation statuses based on the TCGA dataset. ^*^*P* < 0.05, ^**^*P* < 0.01, ^***^*P* < 0.001, and ^****^*P* < 0.0001.

**Figure S2** HMGN2 expression in glioblastoma multiforme cell lines and knockdown or overexpression in glioblastoma multiforme. **(A, B)** HMGN2 expression was measured by qPCR and western blotting in five glioblastoma multiforme cell lines (LN229, U-87 MG, A172, U118, and U251). **(C-H)** HMGN2 expression was detected after HMGN2 knockdown or overexpression using reverse-transcription PCR analysis and western blotting. **(I)** Analysis of cell cycle distribution of LN229 and U-87 MG cells in the NC, scramble, and HMGN2 knockdown groups. The mean percentages of the population in the G2/M phase were plotted. **(J)** Analysis of cell cycle distribution of LN229 and U-87 MG cells in the VC and HMGN2 OE groups. The mean percentages of the population in the G2/M phase were plotted. All data were expressed as mean ± standard deviation. One-way ANOVA and student's *t*-test were performed to analyze significance; ^*^*P* < 0.05, ^**^*P* < 0.01, ^***^*P* < 0.001, and ^****^*P* < 0.0001.

**Figure S3** HMGN2 knockdown inhibits the glioma stem-like cell proliferation. **(A)** HMGN2 expression in TS543 glioma stem-like cells was measured by western blotting. **(B)** CCK-8 analysis of TS543 cells in the NC, scramble, and HMGN2 knockdown groups. **(C)** Analysis of cell cycle distribution of TS543 cells in the NC, scramble, and HMGN2 knockdown groups. The mean percentages of the population in the G2/M phase were plotted. One-way ANOVA was performed to analyze significance; ^*^*P* < 0.05, ^**^*P* < 0.01, ^***^*P* < 0.001, and ^****^*P* < 0.0001.

**Figure S4** HMGN2 regulated cell cycle-related proteins. **(A)** The KEGG and GO enrichment analyses based on the differentially expressed genes from RNA sequencing of LN229 shHMGN2#2 versus scramble groups. **(B)** Left: The mRNA levels of 12 cell cycle-related genes were examined by qPCR in LN229 and U-87 MG cells of the NC, scramble, and HMGN2 knockdown groups; Right: The mRNA levels of cell cycle-related genes were examined by qPCR in LN229 and U-87 MG cells of the NC, Vector, and HMGN2 OE groups. **(C)** Semi-quantitative analysis of the cell cycle-related proteins by GraphPad. **(D)** The levels of cell cycle-related proteins were examined by western blotting in LN229 and U-87 MG cells of the NC, Vector, and HMGN2 OE groups. **(E)** Analysis of cell cycle distribution of LN229 cells in rescue experiments. The mean percentages of the population in the G2/M phase were plotted. One-way ANOVA was performed to analyze significance; ^*^*P* < 0.05, ^**^*P* < 0.01, ^***^*P* < 0.001, and ^****^*P* < 0.0001.

**Figure S5** H3K27ac and H3K9ac binding peaks in the CDC20 and PCNA promoters. **(A–D)** The analysis from CistromeDB (https://cistrome.org) for H3K27ac and H3K9ac binding peaks in the promoter regions of CDC20 and PCNA in different tissues and cells. **(E)** Chromatin immunoprecipitation analysis of H3K9ac and H3K27ac on the PCNA promoter in LN229 and U-87 MG cells with or without HMGN2 knockdown (2% of the input from each group was pulled down and subjected to qPCR). **(F)** The cell cycle distribution was detected by flow cytometry in the NC, HMGN2-3XFlag, and HMGN2-ΔNLS-3XFlag groups. The mean percentages of the population in the G2/M phase were plotted. All data were expressed as mean ± standard deviation. One-way ANOVA and student's *t*-test were performed to analyze significance; ^*^*P* < 0.05, ^**^*P* < 0.01, ^***^*P* < 0.001, and ^****^*P* < 0.0001.
