# Supplementary figures and images for: HMGN2 accelerates the proliferation and cell cycle progression of glioblastoma by regulating CDC20 expression
Source: Genes Dis. 2024 Sep 12;12(3):101433. doi: 10.1016/j.gendis.2024.101433 (PMC11907455; doi:10.1016/j.gendis.2024.101433)

**A**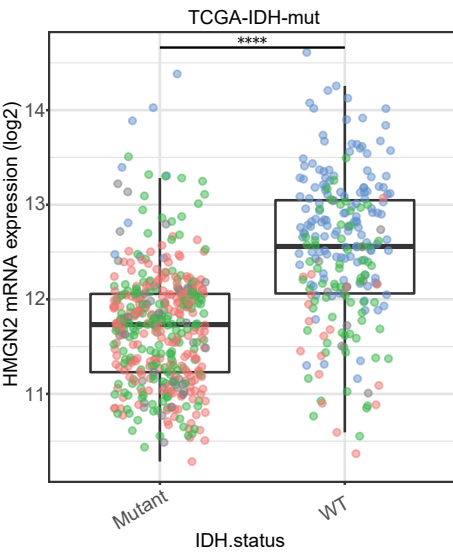**B**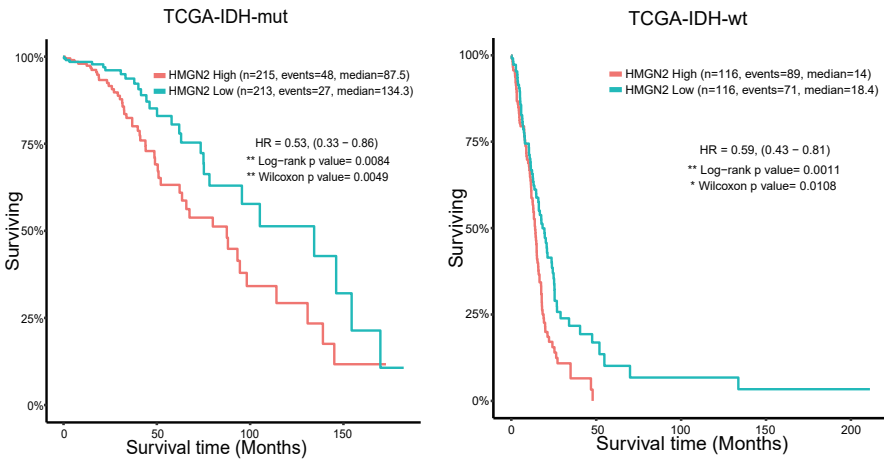**C**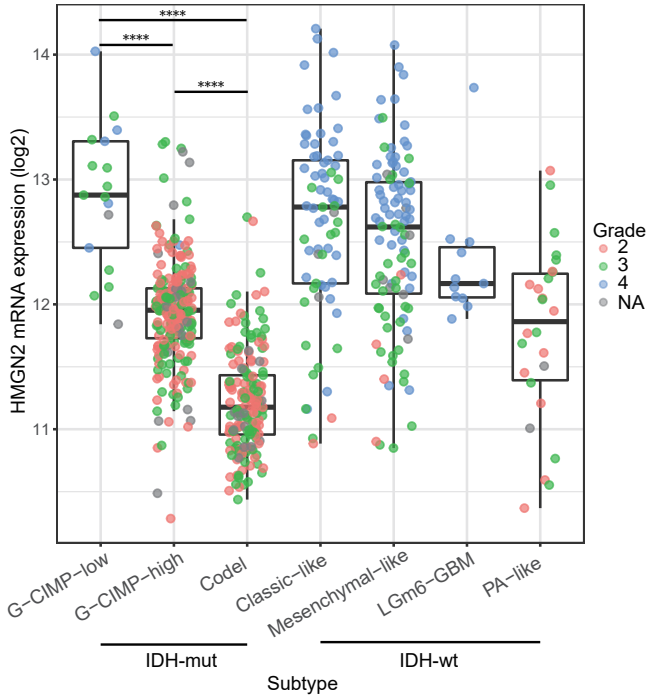**D**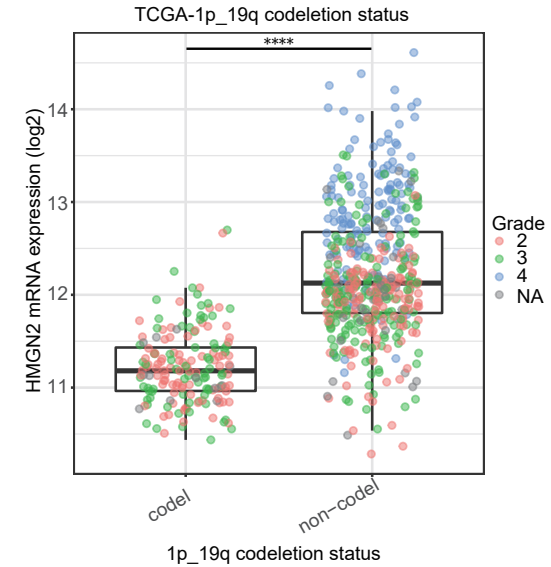**E**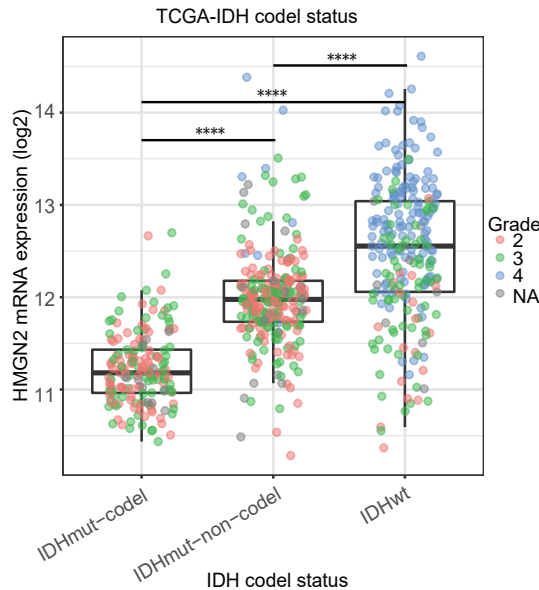**F**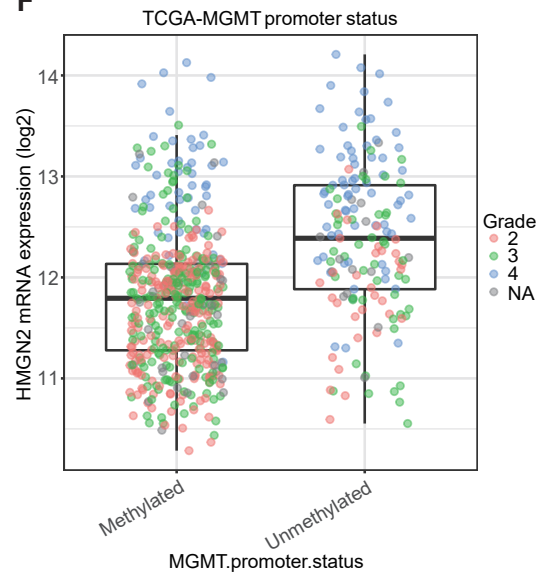

Supplement: Supplementary file 2 — Figure S1. HMGN2 expression across different molecular pathological statuses. (A, B) HMGN2 expression levels in different IDH (isocitrate dehydrogenase) statuses and overall patient survival time across different IDH statuses. (C) Analysis of HMGN2 expression in seven subtypes of glioma based on the TCGA dataset (G-CIMP-low, G-CIMP-high, Codel, Classic-like, Mesenchymal-like, LGm6-GBM, PA-like). (D) HMGN2 expression levels in different 1p 19q codeletion statuses based on the TCGA dataset. (E) HMGN2 expression levels in different IDH and 1p 19q codeletion statuses based on the TCGA dataset. (F) HMGN2 expression levels in different MGMT (O6-methylguanine-DNA-methyltransferase) promoter methylation statuses based on the TCGA dataset. ∗P < 0.05, ∗∗P < 0.01, ∗∗∗P < 0.001, and ∗∗∗∗P < 0.0001. [file mmc2.pdf]

**A**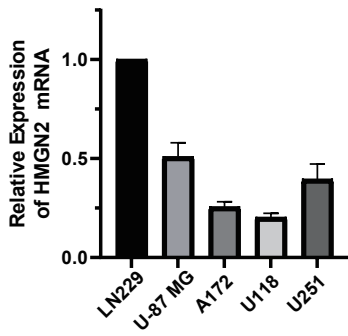**B**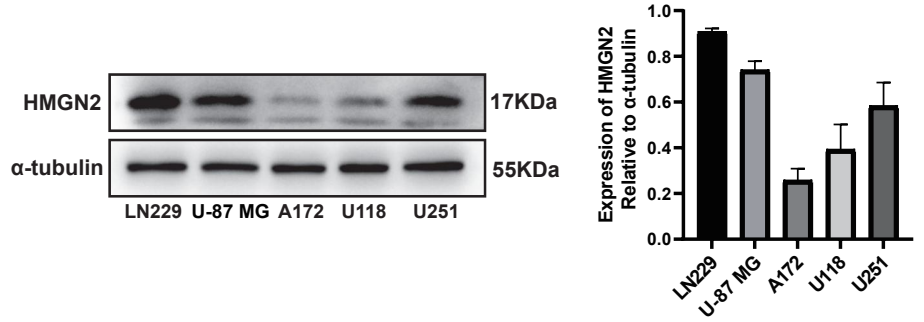**C**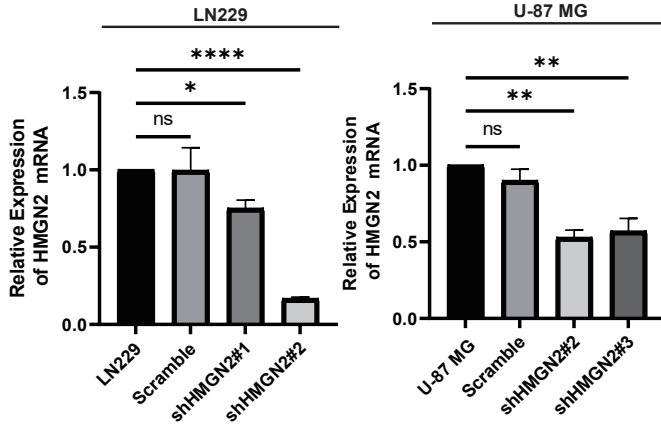**D**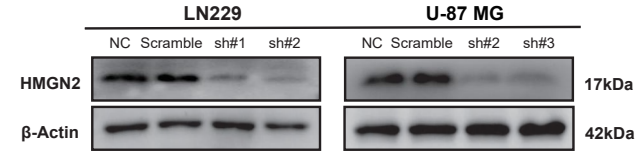**E**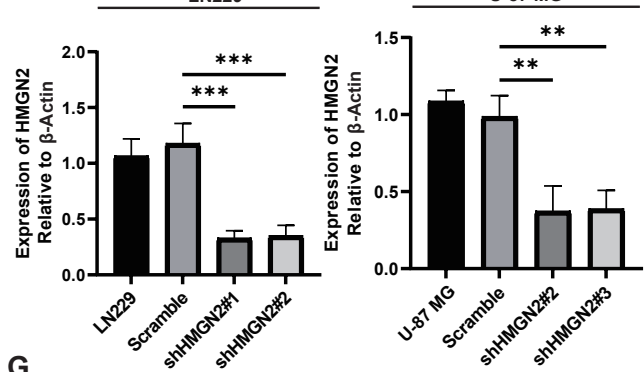**G**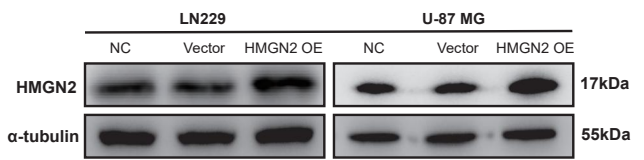**H**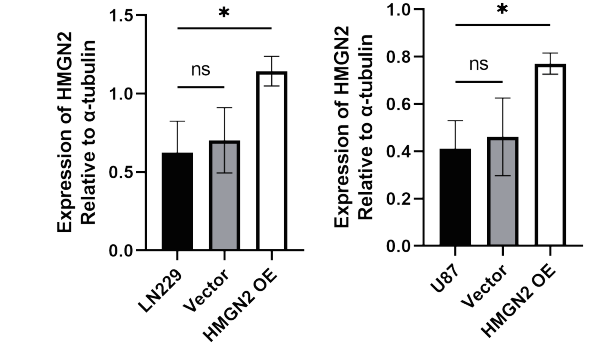**I**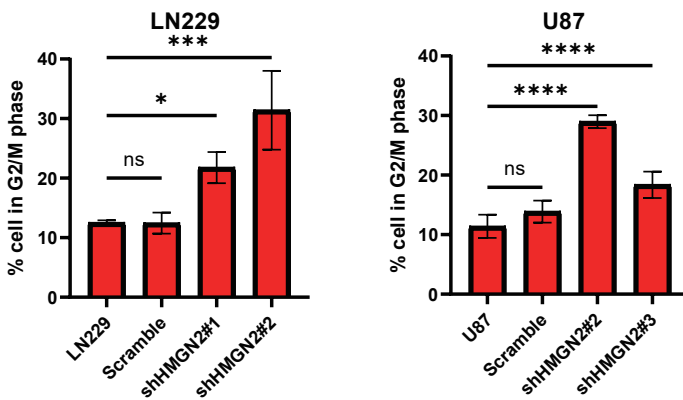**J**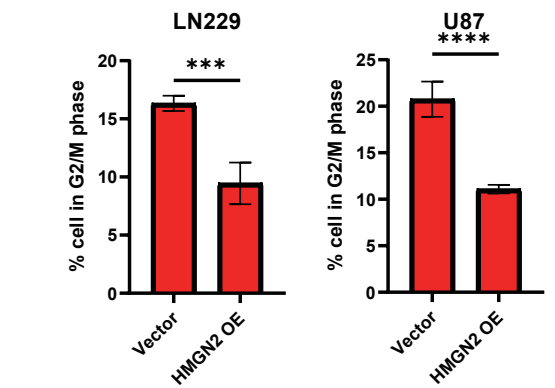

Supplement: Supplementary file 3 — Figure S2. HMGN2 expression in glioblastoma multiforme cell lines and knockdown or overexpression in glioblastoma multiforme. (A, B) HMGN2 expression was measured by qPCR and western blotting in five glioblastoma multiforme cell lines (LN229, U-87 MG, A172, U118, and U251). (C–H) HMGN2 expression was detected after HMGN2 knockdown or overexpression using reverse-transcription PCR analysis and western blotting. (I) Analysis of cell cycle distribution of LN229 and U-87 MG cells in the NC, scramble, and HMGN2 knockdown groups. The mean percentages of the population in the G2/M phase were plotted. (J) Analysis of cell cycle distribution of LN229 and U-87 MG cells in the VC and HMGN2 OE groups. The mean percentages of the population in the G2/M phase were plotted. All data were expressed as mean ± standard deviation. One-way ANOVA and student's t-test were performed to analyze significance; ∗P < 0.05, ∗∗P < 0.01, ∗∗∗P < 0.001, and ∗∗∗∗P < 0.0001. [file mmc3.pdf]

**A**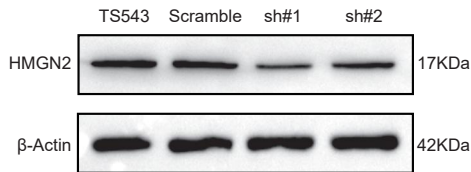**B****TS543**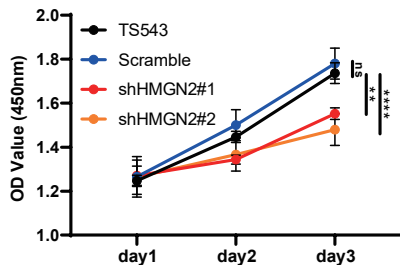**B**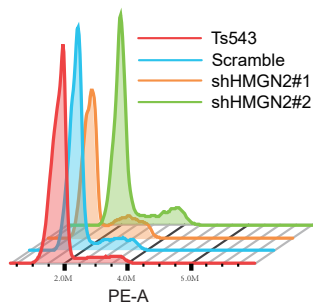**Ts543**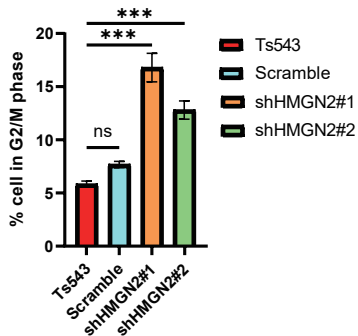

Supplement: Supplementary file 4 — Figure S3. HMGN2 knockdown inhibits the glioma stem-like cell proliferation. (A) HMGN2 expression in TS543 glioma stem-like cells was measured by western blotting. (B) CCK-8 analysis of TS543 cells in the NC, scramble, and HMGN2 knockdown groups. (C) Analysis of cell cycle distribution of TS543 cells in the NC, scramble, and HMGN2 knockdown groups. The mean percentages of the population in the G2/M phase were plotted. One-way ANOVA was performed to analyze significance; ∗P < 0.05, ∗∗P < 0.01, ∗∗∗P < 0.001, and ∗∗∗∗P < 0.0001. [file mmc4.pdf]

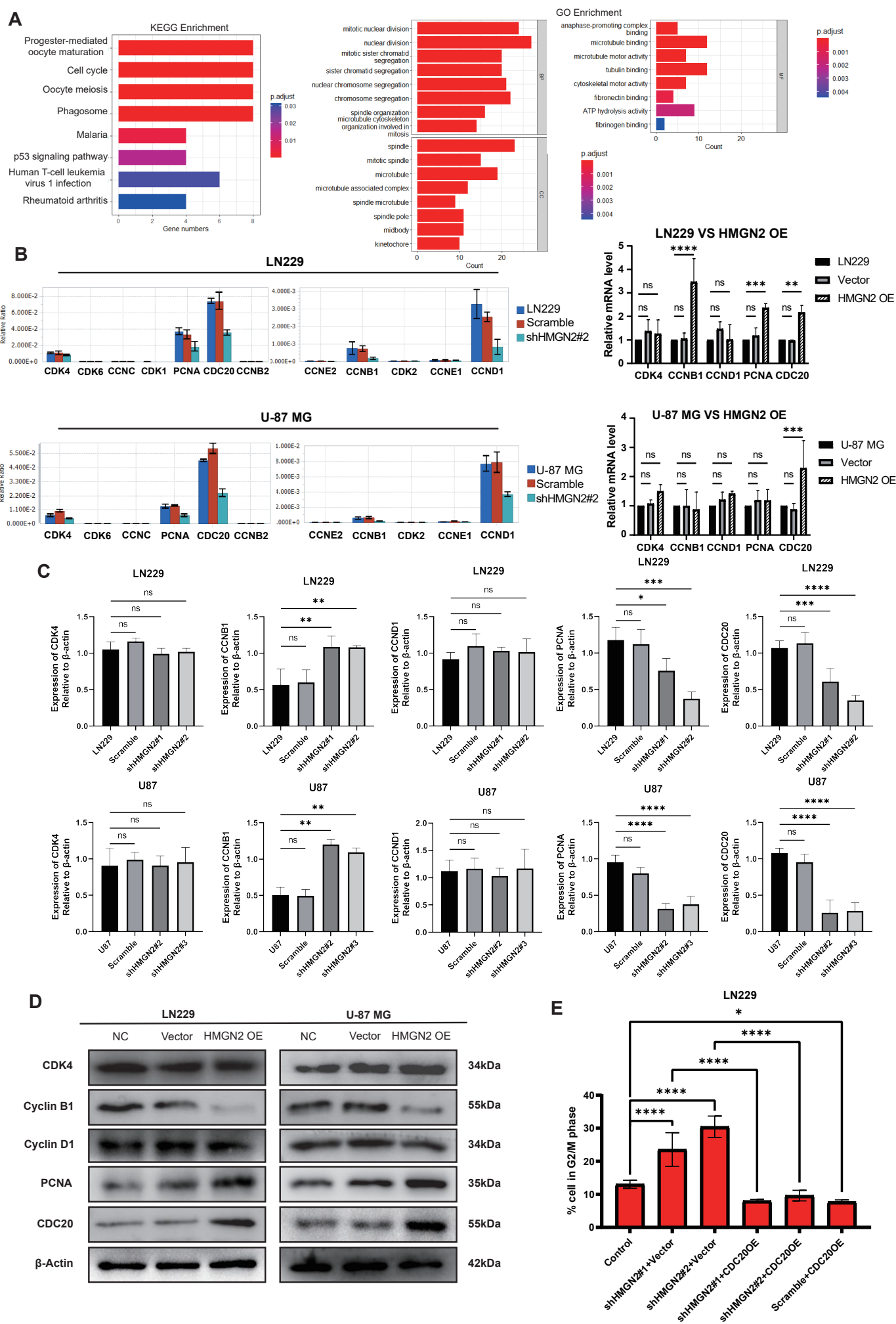

Supplement: Supplementary file 5 — Figure S4. HMGN2 regulated cell cycle-related proteins. (A) The KEGG and GO enrichment analyses based on the differentially expressed genes from RNA sequencing of LN229 shHMGN2#2 versus scramble groups. (B) Left: The mRNA levels of 12 cell cycle-related genes were examined by qPCR in LN229 and U-87 MG cells of the NC, scramble, and HMGN2 knockdown groups; Right: The mRNA levels of cell cycle-related genes were examined by qPCR in LN229 and U-87 MG cells of the NC, Vector, and HMGN2 OE groups. (C) Semi-quantitative analysis of the cell cycle-related proteins by GraphPad. (D) The levels of cell cycle-related proteins were examined by western blotting in LN229 and U-87 MG cells of the NC, Vector, and HMGN2 OE groups. (E) Analysis of cell cycle distribution of LN229 cells in rescue experiments. The mean percentages of the population in the G2/M phase were plotted. One-way ANOVA was performed to analyze significance; ∗P < 0.05, ∗∗P < 0.01, ∗∗∗P < 0.001, and ∗∗∗∗P < 0.0001. [file mmc5.pdf]

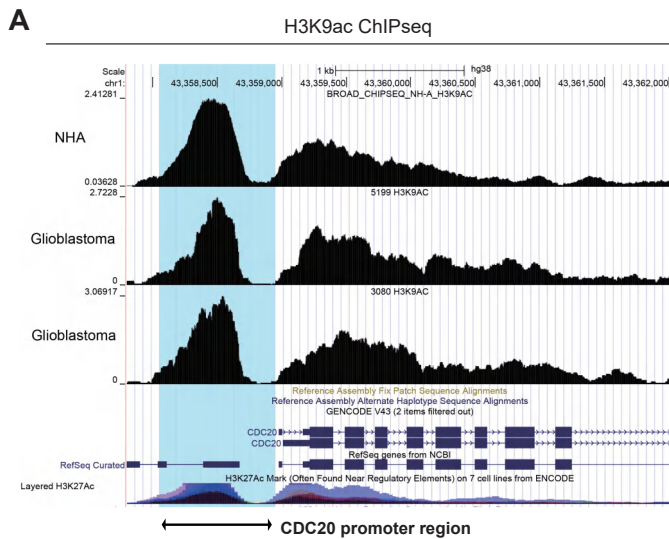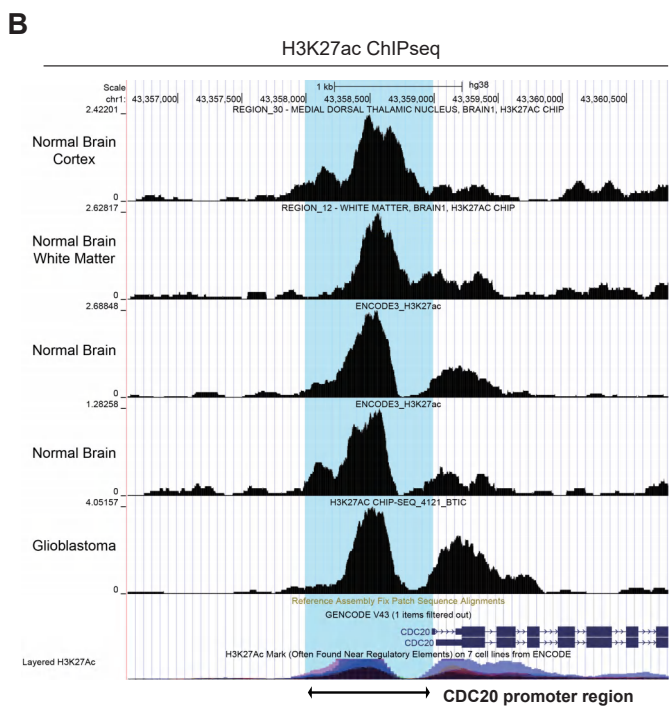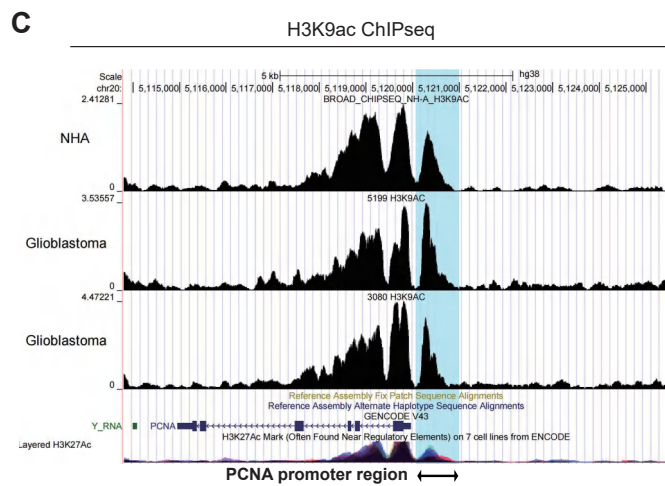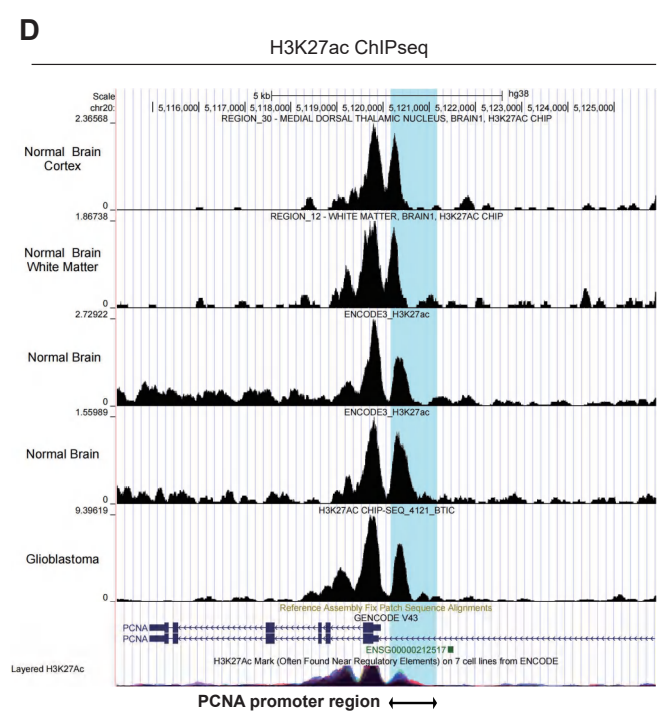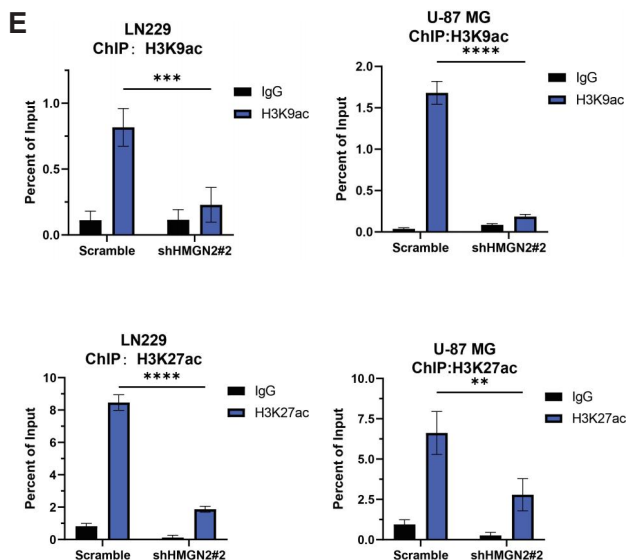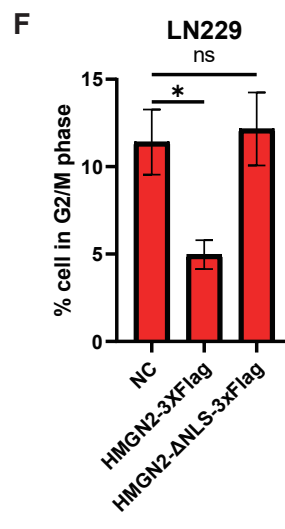

Supplement: Supplementary file 6 — Figure S5. H3K27ac and H3K9ac binding peaks in the CDC20 and PCNA promoters. (A–D) The analysis from CistromeDB (https://cistrome.org) for H3K27ac and H3K9ac binding peaks in the promoter regions of CDC20 and PCNA in different tissues and cells. (E) Chromatin immunoprecipitation analysis of H3K9ac and H3K27ac on the PCNA promoter in LN229 and U-87 MG cells with or without HMGN2 knockdown (2% of the input from each group was pulled down and subjected to qPCR). (F) The cell cycle distribution was detected by flow cytometry in the NC, HMGN2-3XFlag, and HMGN2-ΔNLS-3XFlag groups. The mean percentages of the population in the G2/M phase were plotted. All data were expressed as mean ± standard deviation. One-way ANOVA and student's t-test were performed to analyze significance; ∗P < 0.05, ∗∗P < 0.01, ∗∗∗P < 0.001, and ∗∗∗∗P < 0.0001. [file mmc6.pdf]
